# *Chlamydomonas* IFT25 is dispensable for flagellar assembly but required to export the BBSome from flagella

**DOI:** 10.1242/bio.026278

**Published:** 2017-11-15

**Authors:** Bin Dong, Song Wu, Jing Wang, Yan-Xia Liu, Zhao Peng, De-Mei Meng, Kaiyao Huang, Mingfu Wu, Zhen-Chuan Fan

**Affiliations:** 1 Key Laboratory of Food Nutrition and Safety, Ministry of Education of China, Institute of Health Biotechnology, International Collaborative Research Center for Health Biotechnology, College of Food Engineering and Biotechnology, Tianjin University of Science & Technology, Tianjin 300457, People's Republic of China; 2 Cardiovascular Science Center, Albany Medical College, Albany, New York 12208, USA; 3 Key Laboratory of Algal Biology, Institute of Hydrobiology, Chinese Academy of Sciences, Wuhan, Hubei, 430072, People's Republic of China; 4 Obesita & Algaegen LLC, College Station, Texas 77845, USA

**Keywords:** *Chlamydomonas reinhardtii*, Intraflagellar transport, IFT protein, Flagellar assembly, BBSome

## Abstract

Intraflagellar transport (IFT) particles are composed of polyprotein complexes IFT-A and IFT-B as well as cargo adaptors such as the BBSome. Two IFT-B subunits, IFT25 and IFT27 were found to form a heterodimer, which is essential in exporting the BBSome out of the cilium but not involved in flagellar assembly and cytokinesis in vertebrates. Controversial results were, however, recorded to show that defects in IFT, flagellar assembly and even cytokinesis were caused by IFT27 knockdown in *Chlamydomonas reinhardtii*. Using *C. reinhardtii* as a model organism, we report that depletion of IFT25 has no effect on flagellar assembly and does not affect the entry of the BBSome into the flagellum, but IFT25 depletion did impair BBSome movement out of the flagellum, clarifying the evolutionally conserved role of IFT25 in regulating the exit of the BBSome from the flagellum cross species. Interestingly, depletion of IFT25 causes dramatic reduction of IFT27 as expected, which does not cause defects in flagellar assembly and cytokinesis in *C. reinhardtii*. Our data thus support that *Chlamydomonas* IFT27, like its vertebrate homologues, is not involved in flagellar assembly and cytokinesis.

## Abstract (precis)

**Summary:** Chlamydomonas IFT25 is not involved in flagellar assembly, but is required for export of the BBSome from the flagellum.

## INTRODUCTION

Cilia and flagella project from the surface of most eukaryotic cells in interphase and share the same organelle structure that consists of a microtubule extension called axoneme surrounded by a specialized plasma membrane (Dutcher, 2003; Rosenbaum and Witman, 2002). Although these organelles differ in performing signaling and motility-based functions they are assembled by an evolutionally conserved process called intraflagellar transport (IFT), where the linear IFT trains of large protein complexes undergo robust bidirectional motility along the axoneme (Kozminski et al., 1993; Pigino et al., 2009). During IFT, IFT trains are carried to the flagellar tip by the heterotrimeric kinesin-2 motor and back to the cell body by the cytoplasmic dynein-1b motor (Cole et al., 1998; Pazour et al., 1999, 1998; Porter et al., 1999; Rosenbaum and Witman, 2002). IFT trains are composed of IFT particles and adaptor complexes like the BBSome. IFT particles consist of two different complexes, called IFT-A and IFT-B (Cole et al., 1998; Follit et al., 2009; Ou et al., 2007). The function of IFT-A and -B has been dissected to be responsible for retrograde and anterograde IFT, respectively (Iomini et al., 2001; Piperno et al., 1998; Tsao and Gorovsky, 2008). As a result, mutations in IFT-B subunits and the kinesin motor typically block the anterograde IFT, resulting in loss of flagella (Brazelton et al., 2001; Brown et al., 2015; Deane et al., 2001; Hou et al., 2007; Kobayashi et al., 2007; Miller et al., 2005; Mueller et al., 2005; Pazour et al., 2000); whereas defects in IFT-A subunits and the dynein motors typically hamper the retrograde IFT, leading to the production of stumpy flagella that accumulates materials in flagella (Bizet et al., 2015; Engel et al., 2012; Hou et al., 2004; Iomini et al., 2009; Lin et al., 2013; Niwa, 2016; Pazour et al., 1999, 1998; Piperno et al., 1998; Porter et al., 1999). The BBSome is characteristic of an IFT cargo adaptor and null mutants lacking individual BBS proteins hardly disrupt IFT and flagellar assembly but cause accumulations of abnormal membrane-associated proteins in the flagellum of *C. reinhardtii* (Lechtreck et al., 2009a) or defects in promoting ciliary targeting of membrane proteins in mammals (Berbari et al., 2008; Jin et al., 2010). Although how IFT-A, IFT-B and the BBSome interact to assemble functional IFT trains remains largely unknown, recent studies have shown that the IFT-B subunit IFT74 is required for the coupling between IFT-A and IFT-B, at least in *C. reinhardtii* (Brown et al., 2015), and a second IFT-B subunit, the small GTPase IFT27, plays a role in linking the BBSome to IFT-B as found in the mouse model (Eguether et al., 2014; Liew et al., 2014).

Among the 16 IFT-B particle proteins identified thus far, two IFT-B subunits, IFT25 (Follit et al., 2009; Keady et al., 2012; Lechtreck et al., 2009b; Wang et al., 2009) and the small Rab-like GTPase IFT27 (Qin et al., 2007), are unique in that the two proteins are conserved in vertebrates *C. reinhardtii* and *Trypanosoma* *brucei*, but lack orthologues in some ciliated organisms such as *Caenorhabditis elegans* and *Drosophila* (Aldahmesh et al., 2014; Eguether et al., 2014; Follit et al., 2009; Huet et al., 2014; Iomini et al., 2009; Keady et al., 2012; Lechtreck et al., 2009b; Liew et al., 2014; Qin et al., 2007; Wang et al., 2009). Both proteins differ from other conventional IFT-B subunits in that depletion of either of two proteins in mouse or mammalian cells blocked the export of the BBSome out of the cilium but did not cause defects in flagellar assembly (Eguether et al., 2014; Keady et al., 2012; Liew et al., 2014). This is easy to understand because mammalian IFT25 acts as a binding partner of IFT27 and is essential to maintain the stability of IFT27 (Eguether et al., 2014; Keady et al., 2012; Liew et al., 2014). As a result, knockout of IFT25 resulted in almost complete loss of IFT27 and eventually caused the same phenotype as that caused by IFT27 knockout (Eguether et al., 2014; Keady et al., 2012; Liew et al., 2014). Interestingly, controversial results were recorded in the literature that knockdown of IFT27 caused the dissociation of IFT particles, loss of flagella and even defects in cytokinesis in *C. reinhardtii* (Qin et al., 2007), or led to failure to import IFT-A and IFT dynein into flagella in *T. brucei* (Huet et al., 2014)*.* Although the underlying molecular mechanisms seem different, both cases gained a common outcome that loss of IFT27 causes defects in IFT and flagellar assembly. Taken together, these results suggest that IFT25 and IFT27 probably play a role in IFT and flagellar assembly in a species-dependent manner (Eguether et al., 2014; Huet et al., 2014; Keady et al., 2012; Liew et al., 2014; Qin et al., 2007). *Chlamydomonas* IFT25 was also proven to be essential to maintain the stability of IFT27 (Bhogaraju et al., 2011) which is supposed to cause the same defects in IFT, flagellar assembly and cytokinesis as that caused by IFT27 knockdown (Qin et al., 2007), and depletion of *Chlamydomonas* IFT25 thus is supposed to cause depletion of IFT27. However, it was noted that the specificity of the IFT27 knockdown phenotype was not proven in *C. reinhardtii* in the previous study, as a strict functional rescue assay was not performed and an off-target effect thus cannot be excluded (Qin et al., 2007). Therefore, for the first time, we aim to clarify the role of IFT25 in IFT and flagellar assembly in *C. reinhardtii* to investigate if the role of IFT25 in IFT and flagellar assembly was evolutionally conserved or species specific.

## RESULTS

### IFT25 has a similar cellular distribution pattern as IFT proteins and undergoes IFT in flagella

**Fig. 1.**
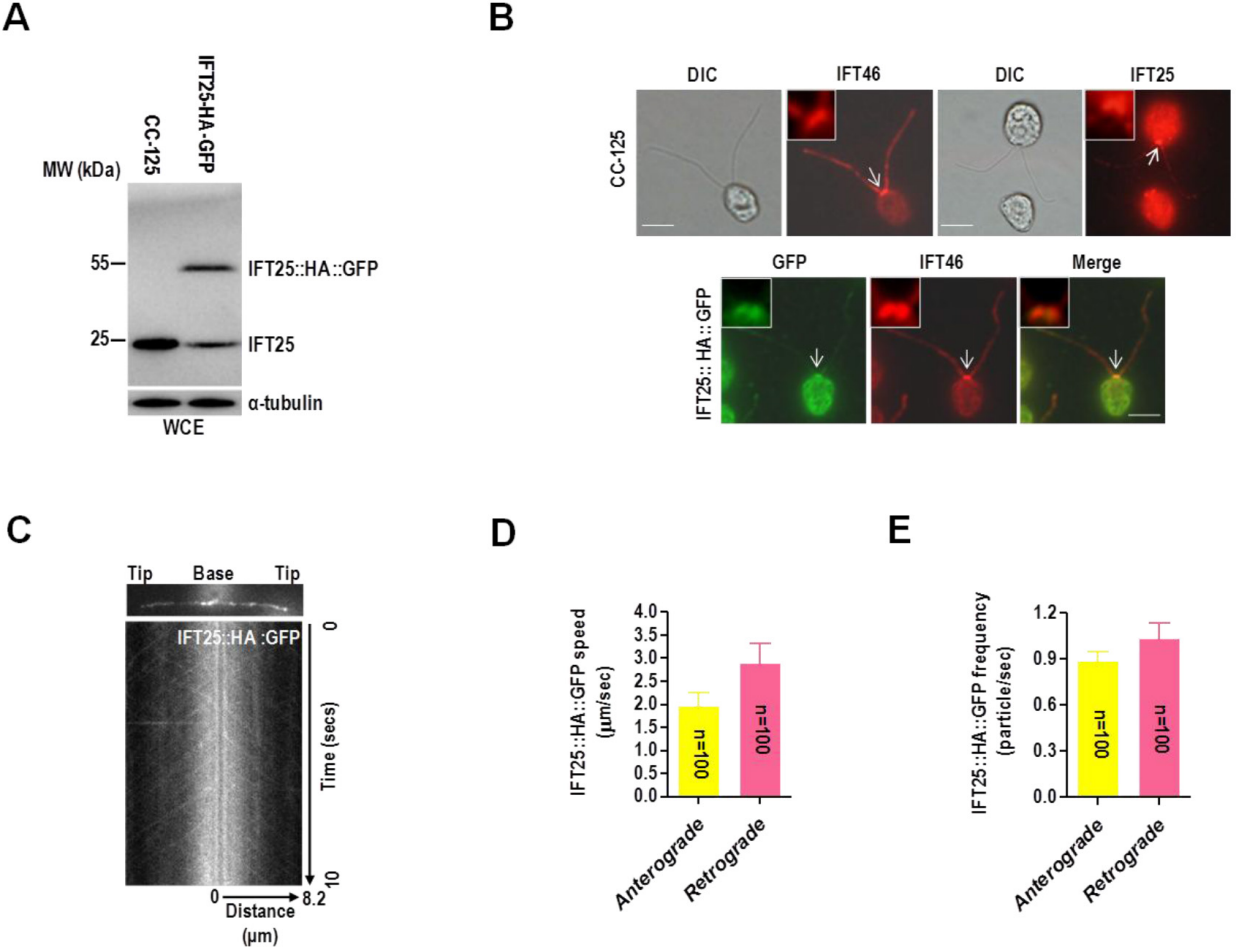
**IFT25 localizes in the basal body and flagella and undergoes IFT in flagella.** (A) Western blots of wild-type CC-125 cells and a GFP-expressing CC-125 transgenic strain with antibodies to IFT25 and α-tubulin. WCE, whole cell extracts. (B) Immunofluorescence images of wild-type CC-125 and IFT25::HA::GFP strains. As detected in wild-type CC-125 cells, IFT25 has a similar basal body and flagella localization pattern as IFT46 (upper panel). When checked in the IFT25-HA-GFP transgenic cells, IFT25-HA-GFP and IFT46 co-localized at the basal bodies and flagella (lower panel) (green, α-GFP; red, α-IFT25 and α-IFT46). DIC, phase contrast photos. White arrows indicate the basal body to be enlarged in the top-left corner of each picture. Scale bars: 5 µm. (C) Single frame and kymograph from TIRF microscopy video imaging of IFT25::HA::GFP transgenic strain (Movie 1, ∼10 fps). (D) The speeds of anterograde and retrograde IFT25::HA::GFP in the IFT25::HA::GFP transgenic strain. Anterograde IFT25::HA::GFP speed is 1.97±0.28 μm/s (*n*=100, *P*=0.037) and retrograde IFT25::HA::GFP speed is 2.90±0.43 μm/s (*n*=100, *P*=0.052). Speeds are mean values±95% confidence interval and *P*-values were calculated from a student's *t*-test. (E) The frequencies of anterograde and retrograde IFT25::HA::GFP in the IFT25::HA::GFP transgenic strain. The anterograde frequency of IFT25::HA::GFP is 0.89±0.06 particle/sec (*n*=100, *P*=0.042) and its retrograde frequency is 1.04±0.10 particle/sec (*n*=100, *P*=0.028). Frequencies are mean values±95% confidence interval and *P*-values were calculated from a student's *t*-test. In D and E, data are presented as mean±s.d.

Our previous study showed that IFT25 has spotted distribution along the flagellum, thus showing as a typical pattern of IFT protein (Wang et al., 2009). However, its localization pattern in the basal body is unique in that IFT25 sits right above the basal body but not inside of it, as shown by other IFT-B subunits (Wang et al., 2009). This was probably caused by the poor quality of the anti-IFT25 antibody used. In this study, a full-length *C. reinhardtii* IFT25 was expressed, purified, and used to produce antisera in two rabbits (Fig. S1A,B). Western blotting assay showed that the anti-IFT25 antibody detected one single band of approximately 20 kDa in wild-type cells (Fig. 1A). One additional fusion protein band with a predicted size of approximately 50 kDa was also detected by the same antibody in a transgenic strain expressing IFT25::HA::GFP (Fig. 1A). The transgenic stain remained totally IFT25 (the native IFT25 plus the IFT25::HA::GFP fusion protein), the same as wild-type cells, indicating that the level of IFT25 in wild-type cells is probably tightly controlled.

In wild-type cells, the anti-IFT25 antibody detected signals along the flagellum (Fig. 1B). At the basal body region, IFT25 had a distribution pattern similar to IFT46 (Fig. S2A,B for anti-IFT46 antiserum generation) as a band perpendicular to each basal body (Fig. 1B). To confirm this observation, the transgenic strain expressing IFT25::HA::GFP was applied to determine the cellular localization pattern of the native IFT25 by using a monoclonal anti-GFP antibody. Immunofluorescence staining showed that IFT25::HA::GFP exhibited a similar localization as IFT46, with the presence of the proteins in the basal body and inside flagella (Fig. 1B). As a result, both the native and fusion IFT25 proteins thus showed typical IFT protein distribution pattern in the basal body and flagella. In addition, total internal reflection fluorescence microscopy (TIRFM) analysis performed on living IFT25::HA::GFP transgenic cells showed that IFT25::HA::GFP underwent typical bidirectional IFT (Movie 1), similar to IFT traffick­ing observed for *C. reinhardtii* IFT27::GFP (Qin et al., 2007) and KAP (Mueller et al., 2005). Once kymographs (Fig. 1C) were made to quantify IFT train speed the mean anterograde speed was determined to be 1.97±0.28 μm/s (*n*=100) and mean retrograde speed was measured at 2.90±0.43 μm/s (*n*=100) (Fig. 1D). In addition, the mean frequencies of anterograde and retrograde IFT25::HA::GFP were measured to be 0.89±0.06 particle/sec (*n*=100) and 1.04±0.10 particle/sec (*n*=100), respectively, also similar to the frequencies observed for IFT56-GFP (Ishikawa et al., 2014) (Fig. 1E). Overall, these results show that IFT25 distribution and movement in flagella are similar to the other IFT proteins studied to date in *C. reinhardtii*, and the N-terminal GFP-tagged IFT25 was functional in IFT and flagellar assembly and can be used to replace the native IFT25.

### IFT25 and IFT27 form a heterodimer to exist both in IFT-B and outside of it *in vivo*

**Fig. 2.**
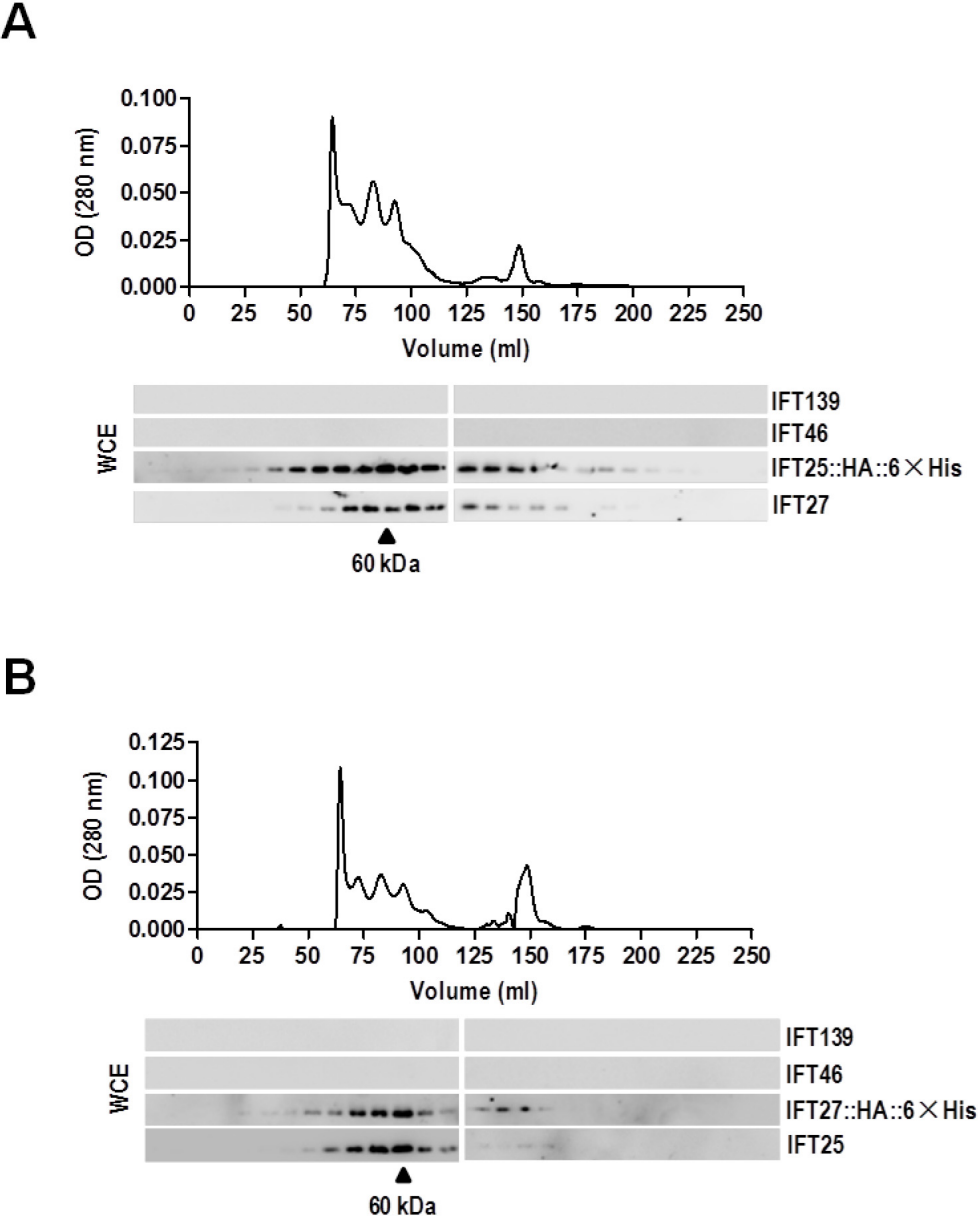
**IFT25 and IFT27 form an IFT-A- and IFT-B-independent heterodimer *in vivo*.** Ni-NTA resin elute from whole cell lysates of the IFT25::HA::6×His (A) and IFT27::HA::6×His (B) transgenic strains were fractionated by size exclusion chromatography (S200 sizing column). As detected by western blotting, IFT25::HA::6×His and IFT27 cofractionated at ∼60 kDa, independent of IFT-A protein IFT139 and IFT-B protein IFT46 (A) and IFT27::HA::6×His and IFT25 also cofractionated at ∼60 kDa, independent of IFT139 and IFT46 (B). For both panels, relative total protein concentration of the fractions is shown as absorbance at 280 nm. The elution volume of one standard marker (60 kDa) is indicated on the western blots. WCE, whole cell extracts.

Our previous study had shown that IFT25 and IFT27 co-sediment with IFT-B proteins in sucrose density gradients of both the whole cell and flagellar extracts of *C. reinhardtii* (Wang et al., 2009). Different from other IFT-B complex proteins, the majority of IFT25 and IFT27 are also observed to appear in a discrete IFT25- and IFT27-containing small peak in sucrose density gradients of both whole cell and flagellar extracts of *C. reinhardtii*, and the two proteins physically interact with each other (Wang et al., 2009). To determine if there were any other components than IFT25 and IFT27 in the small complex *in vivo*, we purified IFT25- and IFT27-associated protein complexes from whole cell extracts of two transgenic strains expressing either IFT27::HA::6×His or IFT25::HA::6×His. To detect the appearance of IFT27 in the complex by western blotting assay, the N-terminal 18 amino acids of *C. reinhardtii* IFT27 were synthesized and used to produce antisera in two rabbits (Fig. S3). Gel filtration chromatography of the eluate from the Ni-NTA resin showed cofractionation of IFT27::HA::6×His and IFT25 or IFT25::HA::6×His and IFT27 with a molecular weight of 60 kDa, indicating that the small complex only contains IFT25 and IFT27 as its protein components (Fig. 2A,B). Based on this observation, we conclude, along with our previous result (Wang et al., 2009), that IFT25 and IFT27 form a heterodimeric complex we called IFT25/27 that exists both within the IFT-B complex and outside of it *in vivo*.

### IFT25 does not affect the stability of IFT-A and -B complexes

**Fig. 3.**
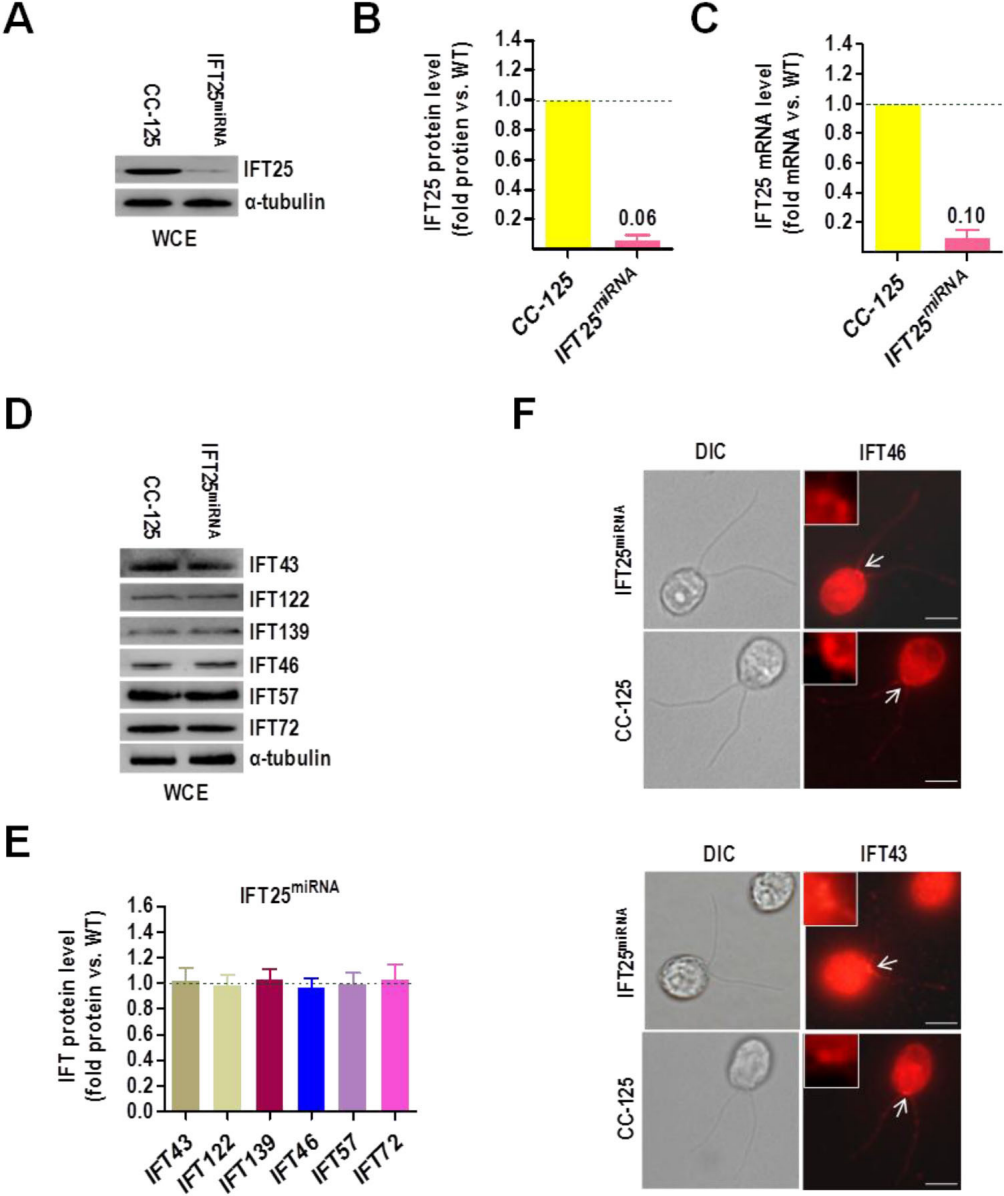
**IFT25 does not affect the stability of IFT-A and -B complexes.** (A) Western blots comparing the cellular levels of IFT25 in wild-type CC-125 and IFT25^miRNA^ cells. Alpha-tubulin was used as a loading control. WCE, whole cell extracts. (B) IFT25 protein levels were normalized to the α-tubulin housekeeping protein and presented as percentage-change relative to wild-type CC-125 IFT25. The data shown was calculated from three western blots (*n*=3 repeats). Error bars indicate s.d. As calculated, IFT25^miRNA^ cells contained approximately 6% as much IFT25 as CC-125 cells. (C) Real-time PCR comparing the IFT25 mRNA in wild-type CC-125 and IFT25^miRNA^ cells. Transcript levels were normalized to the GBLP housekeeping gene and presented as percentage-change relative to wild-type CC-125 mRNA. The data shown was calculated from three independent assays (*n*=3 repeats). Error bars indicate s.d. As calculated, IFT25^miRNA^ cells contained approximately 10% as much IFT25 mRNA as CC-125 cells. (D) Western blots of wild-type CC-125 and IFT25^miRNA^ whole cell extracts with antibodies against the IFT-A proteins IFT43, IFT122 and IFT139 and the IFT-B proteins IFT46, IFT57 and IFT72 as indicated. Alpha-tubulin was used as a loading control. WCE, whole cell extracts. (E) Protein levels of the IFT-A proteins IFT43, IFT122 and IFT139 and the IFT-B proteins IFT46, IFT57 and IFT72 were normalized to the α-tubulin housekeeping protein and presented as percentage-change relative to wild-type CC-125 counterparts. As calculated, both IFT-A and IFT-B components remained unchanged in IFT25^miRNA^ as compared to CC-125 cells. The data shown was calculated from three western blots (*n*=3 repeats). Error bars indicate s.d. (F) Immunofluorescence images of wild-type CC-125 and IFT25^miRNA^ cells. As detected in both wild-type CC-125 and IFT25^miRNA^ cells, the IFT-A protein IFT43 and the IFT-B protein IFT46 both show a typical basal body and flagella localization pattern. DIC, phase contrast photos. White arrows indicate the basal body to be enlarged in the top-left corner of each picture. Scale bars: 5 µm.

Each IFT-B complex protein contributes to the stability of IFT-B complex. Loss of a single IFT-B complex protein typically causes partial or complete dissociation of IFT-B complex, depending on which protein is depleted (Brazelton et al., 2001; Fan et al., 2010; Hou et al., 2007; Pazour et al., 2000; Qin et al., 2007). To date, IFT27 is the only IFT-B subunit that, once partially depleted by interfering RNA technology, was shown to cause dissociation of both IFT-A and -B complexes in *C. reinhardtii* (Qin et al., 2007). To test whether IFT25 influences the stability of IFT-A and -B complexes, we created a vector-based IFT25 miRNA strain called IFT25^miRNA^ (Fig. 3A) and measured the protein level of the IFT-B components IFT46, IFT57 (Fig. S4A,B for anti-IFT57 antiserum generation) and IFT72 (Fig. S5A,B for anti-IFT72 antiserum generation) and the IFT-A components IFT43, IFT122 (Fig. S6 for anti-IFT122 antiserum generation) and IFT139 (Fig. S7A,B for anti-IFT139 antiserum generation) in these cells. Western blotting assay showed that IFT25^miRNA^ cells experienced reductions of IFT25 to **∼**6% of wild-type level (Fig. 3B). The IFT25 mRNA of IFT25^miRNA^ cells was reduced to ∼10% of wild type (Fig. 3C), indicating that depletion of IFT25 protein was caused by miRNA interference. However, IFT25^miRNA^ cells did not see a significant depletion of IFT-A and -B proteins (Fig. 3D,E), revealing that IFT25 has no influence on the stability of IFT-A and -B complexes, thus consistent with the observation that knockout of IFT25 in mouse largely does not affect the stability of IFT particle proteins (Eguether et al., 2014; Keady et al., 2012). In addition, the IFT-A protein IFT43 and the IFT-B protein IFT46 of the IFT25^miRNA^ cells, similar to that of the wild-type CC-125 cells, showed typical basal body and flagellar distribution patterns, suggesting that IFT25 depletion does not affect the localization of the IFT-A and -B complexes (Fig. 3F).

### IFT25 is essential to maintain IFT27 stability *in vivo*

**Fig. 4.**
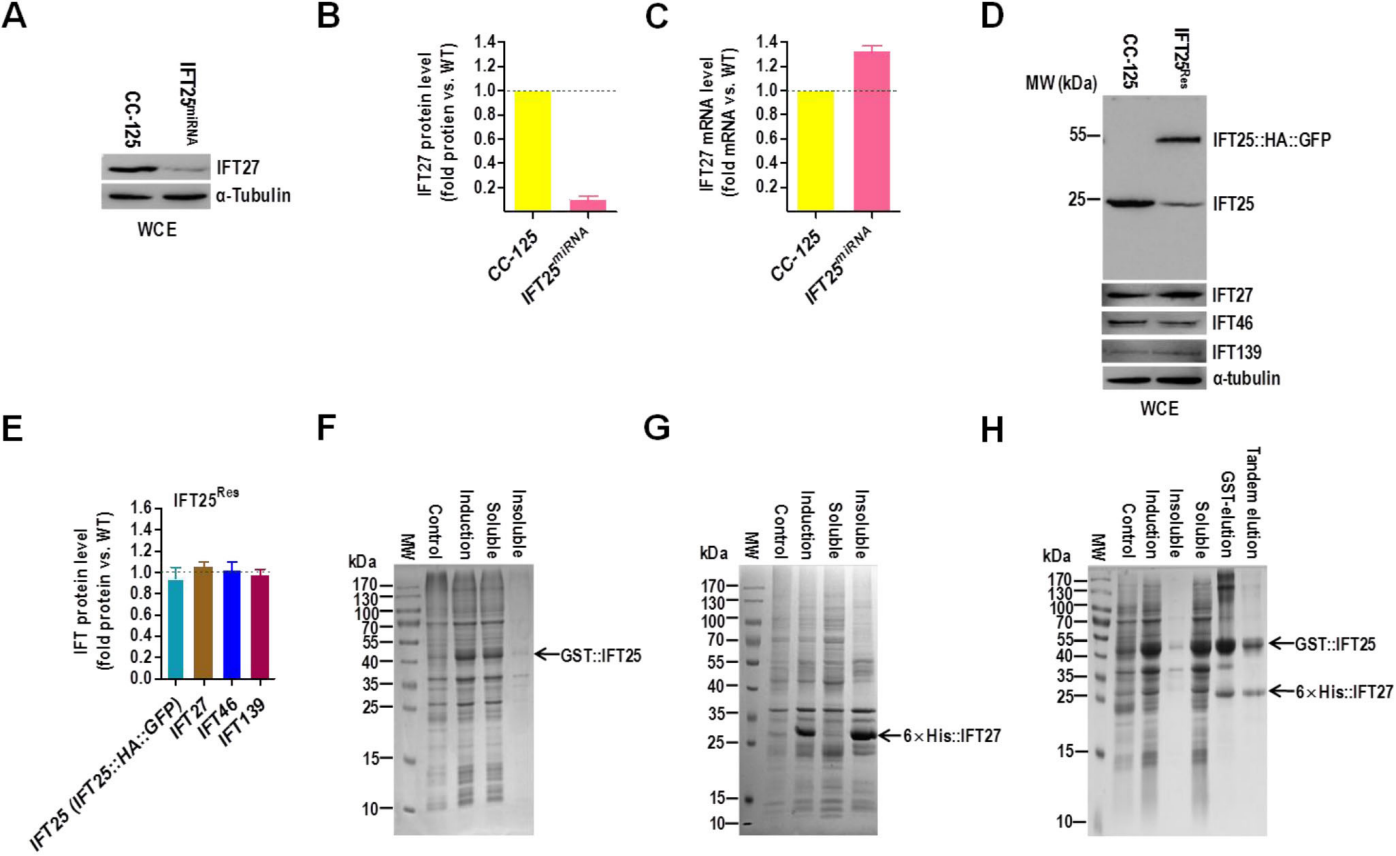
**IFT25 is essential to maintain IFT27 stability *in vivo.*** (A) Western blots comparing the cellular levels of IFT27 in wild-type CC-125 and IFT25^miRNA^ cells. (B) IFT27 protein levels were normalized to the housekeeping protein α-tubulin and presented as percentage-change relative to wild-type CC-125 IFT27. The data shown was calculated from three independent assays (*n*=3 repeats). Error bars indicate s.d. As calculated, IFT25^miRNA^ cells contained approximately 7% as much IFT27 as CC-125 cells. (C) Real-time PCR comparing the IFT27 mRNA in wild-type CC-125 and IFT25^miRNA^ cells. Transcript levels were normalized to the GBLP housekeeping gene and presented as percentage-change relative to wild-type CC-125 mRNA. As calculated, IFT25^miRNA^ cells contained approximately 133% as much IFT27 mRNA as CC-125 cells. The data shown was calculated from three independent assays (*n*=3 repeats). Error bars indicate s.d. (D) Western blots of wild-type CC-125 and IFT25^Res^ whole cell extracts with antibodies to IFT25, IFT27, IFT46 and IFT139. (E) IFT proteins including IFT25, IFT27, IFT46 and IFT139 in IFT25^Res^ cells were normalized to the housekeeping protein α-tubulin and presented as percentage-change relative to their wild-type CC-125 counterparts. When IFT25 protein (IFT25 plus IFT25::HA::GFP) level was rescued in the IFT25^Res^ strain IFT27 level was also rescued in these cells. IFT46 and IFT139 remained unchanged in IFT25^Res^ cells as compared to CC-125. The data shown was calculated from three western blots (*n*=3 repeats). Error bars indicate s.d. (F-H) Coomassie Blue-stained gels, showing that bacterial-expressed recombinant GST::IFT25 is mostly soluble (F), bacterial-expressed recombinant 6×His::IFT27 is mostly insoluble (G) and co-expression of 6×His::IFT27 and GST::IFT25 is primarily present in the soluble fraction (H). For all panels, the lanes indicated as ‘Control’ and ‘Induction'contained the bacterial lysates before and after IPTG induction, respectively. The lanes labeled with ‘Insoluble’ and ‘Soluble’ are the insoluble and soluble fractions recovered after centrifugation of cell lysates. The soluble fractions were further purified by glutathione-agarose resin (F), Ni-NTA resin (G) or ‘tandem’ purification with both resins (H). For panels A and D, alpha-tubulin was used as a western blot loading control. WCE, whole cell extract.

To investigate the influence of IFT25 on IFT27 stability, we measured the protein and transcript levels of IFT27 in the vector-based IFT25 miRNA strain (IFT25^miRNA^). IFT25 depletion in IFT25^miRNA^ cells caused a dramatic reduction of IFT27 protein to a level of as low as **∼**7% of wild-type level (Fig. 4A,B), and a significant increase in IFT27 mRNA to a level of as high as ∼133% of wild-type level (Fig. 4C), indicating that cells respond to the loss of IFT27 protein by increasing IFT27 transcript level. In addition, expression of the IFT25::HA::GFP transgene in IFT25^miRNA^ cells did not cause change on IFT46 and IFT139 protein levels (strain IFT25^Res^) but IFT27 protein abundance was restored upon rescue of IFT25 (IFT25::HA::GFP) expression in the IFT25^Res^ strain (Fig. 4D,E**)**. After continuously passaging IFT25^miRNA^ for six months, the recovered IFT25^Rec^ strain maintained wild-type levels of IFT25, IFT46 and IFT139 proteins as well as IFT27 (Fig. S8A,B). Thus, the stability of IFT27 protein strictly relies on its partner protein IFT25 *in vivo*. By performing an *in vitro* experiment, we found that an N-terminal GST-tagged IFT25 (GST::IFT25) was completely soluble when expressed in bacteria alone (Fig. 4F). However, N-terminal 6×His-tagged IFT27 (6×His::IFT27) was almost completely insoluble when expressed alone in bacteria (Fig. 4G). In contrast, when 6×His::IFT27 and GST::IFT25 were co-expressed in bacteria, the majority of both proteins were present in the soluble fraction after tandem purification (Fig. 4H). By having these *in vitro* and *in vivo* results, we therefore conclude that IFT25 is an obligate binding partner of IFT27 and protects IFT27 from aggregation through a direct interaction (also see Bhogaraju et al., 2011).

### IFT25 is not required for flagellar assembly

**Fig. 5.**
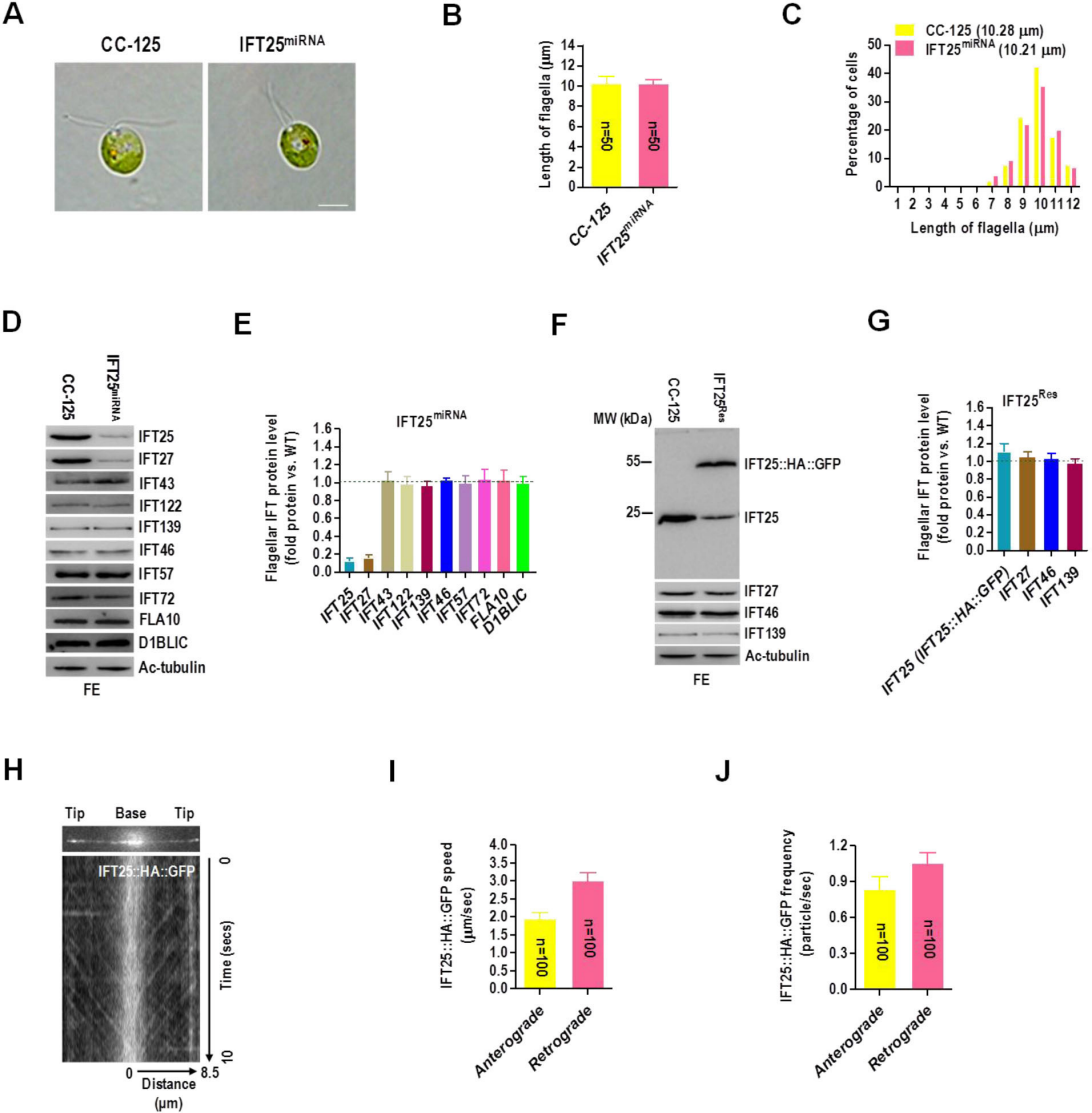
***C. reinhardtii* IFT25 is not required for flagella assembly.** (A) DIC images of wild-type CC-125 and IFT25^miRNA^ cells. Scale bars: 5 µm. (B) CC-125 and IFT25^miRNA^ cells had a mean flagella length of 10.28 µm and 10.21 µm, respectively. The data shown was calculated from 50 flagella (*n*=50 repeats). Error bars indicate s.d. (C) Histograms showing that IFT25^miRNA^ cells had a similar flagella length distribution pattern as CC-125 strain. (D) Western blots of wild-type CC-125 and IFT25^miRNA^ flagellar extracts with antibodies listed on the right. (E) The flagella of IFT25^miRNA^ cells contained approximately 13% and 17% as much IFT25 and IFT27 as flagella of CC-125 cells. (F) Western blots of wild-type CC-125 cells and IFT25^Res^ flagella extracts with antibodies listed on the right. (G) When IFT25 protein (IFT25 plus IFT25::HA::GFP) level was rescued in the IFT25^Res^ strain IFT27 level was also rescued in these cells. IFT46 and IFT139 remained unchanged in IFT25^Res^ flagella as compared to CC-125. (H) Single frame and kymograph from TIRF microscopy video imaging of IFT25^Res^ transgenic strain (Movie 2, ∼10 fps). (I) The speeds of anterograde and retrograde IFT25::HA::GFP in the IFT25::HA::GFP transgenic strain. Anterograde IFT25::HA::GFP speed is 1.93±0.19 μm/s (*n*=100, *P*=0.033) and retrograde IFT25::HA::GFP speed is 3.01±0.23 μm/s (*n*=100, *P*=0.025). Speeds are mean values±95% confidence interval and *P*-values were calculated from a student's *t*-test. (J) The frequencies of anterograde and retrograde IFT25::HA::GFP in the IFT25^Res^ transgenic strain. The anterograde frequency of IFT25::HA::GFP is 0.83±0.11 particle/sec (*n*=100, *P*=0.057) and its retrograde frequency is and 1.05±0.09 particle/sec (*n*=100, *P*=0.089). Frequencies are mean values±95% confidence interval and *P*-values were calculated from a student's *t*-test. For panels D and F, acetylated α-tubulin (Ac-tubulin) was used as a western blot loading control. FE, flagellar extract. For panels E and G, IFT proteins including IFT25, IFT27, the IFT-B component IFT46 and the IFT-A component IFT139 in IFT25^Res^ flagella were normalized to the Ac-tubulin housekeeping protein and presented as percentage-change relative to their wild-type CC-125 counterparts. IFT46 and IFT139 remained unchanged in flagella of IFT25^res^ cells as compared to that of CC-125. The data shown was calculated from three western blots (*n*=3 repeats). Error bars indicate s.d.

Given that depletion of IFT25 has no effect on IFT-A and -B stability, we wondered whether depletion of IFT25 could cause defects in IFT and flagellar assembly. To test this hypothesis, we examined the IFT25^miRNA^ knockdown strain and found that the majority of IFT25^miRNA^ cells were individual cells with wild-type swimming behavior (Fig. 5A). IFT25^miRNA^ cells had flagella with normal length (mean length=10.21 µm) as compared to the wild-type cells (mean length=10.28 µm) (Fig. 5B) and the flagella length distribution of IFT25^miRNA^ cells was also similar to that of CC-125 cells (Fig. 5C). We next checked the levels of IFT25 and IFT27 as well as the IFT-A proteins IFT43, IFT122 and IFT139, the IFT-B proteins IFT46, IFT57 and IFT72, the kinesin subunit FLA10 (Fig. S9 for anti-FLA10 antiserum generation) and the dynein subunit D1BLIC (Fig. S10 for anti-D1BLIC antiserum generation) in flagella isolated from IFT25^miRNA^ cells. When gel loading was normalized for equal Ac-tubulin, the strain showed the same levels of IFT-A, IFT-B and motor proteins as wild-type flagella (Fig. 5D,E). As expected, the amount of IFT25 and IFT27 both dramatically decreased in IFT25^miRNA^ flagella to a level of as low as ∼13% and 17% of wild-type level (Fig. 5D,E). Since the C-terminal HA- and GFP-tagged IFT25 (IFT25::HA::GFP) was functional to participate in IFT (Fig. 1), IFT25::HA::GFP was then used to rescue the authentic IFT25 in IFT25^Res^ strain. When IFT25 (IFT25::HA::GFP) protein level was rescued in the IFT25^miRNA^ strain, the levels of IFT25 (IFT25::HA::GFP) and IFT27 in IFT25^Res^ flagella were determined to be the same as that of the wild-type flagella (Fig. 5F,G). As expected, the recovered strain IFT25^Rec^ maintained wild-type level of IFT25, IFT46 and IFT139 proteins as well as IFT27 in flagella (Fig. S11A,B). TIRFM analysis performed on living IFT25^Res^ cells showed that IFT25::HA::GFP underwent bidirectional IFT movements similar to that in the IFT25::HA::GFP transgenic cells (see Movie 2). After the kymograph was made to quantify IFT train speed (Fig. 5H) the mean anterograde speed was measured at 1.93±0.19 μm/s (*n*=100) and mean retrograde speed was calculated as 3.01±0.23 μm/s (*n*=100) (Fig. 5I). The mean frequencies of anterograde and retrograde IFT25::HA::GFP were measured to be 0.83±0.11 particle/sec and 1.05±0.09 particle/sec, respectively (Fig. 5J), also similar to the frequencies observed for IFT25::HA::GFP under a CC-125 background (Fig. 1E). These observations showed that depletion of IFT25 does not affect IFT protein content (except for IFT27) in flagella and flagella length, suggesting that IFT25 is not required for flagellar assembly in *C. reinhardtii*.

### IFT25 is required for the export of the BBSome from flagella

**Fig. 6.**
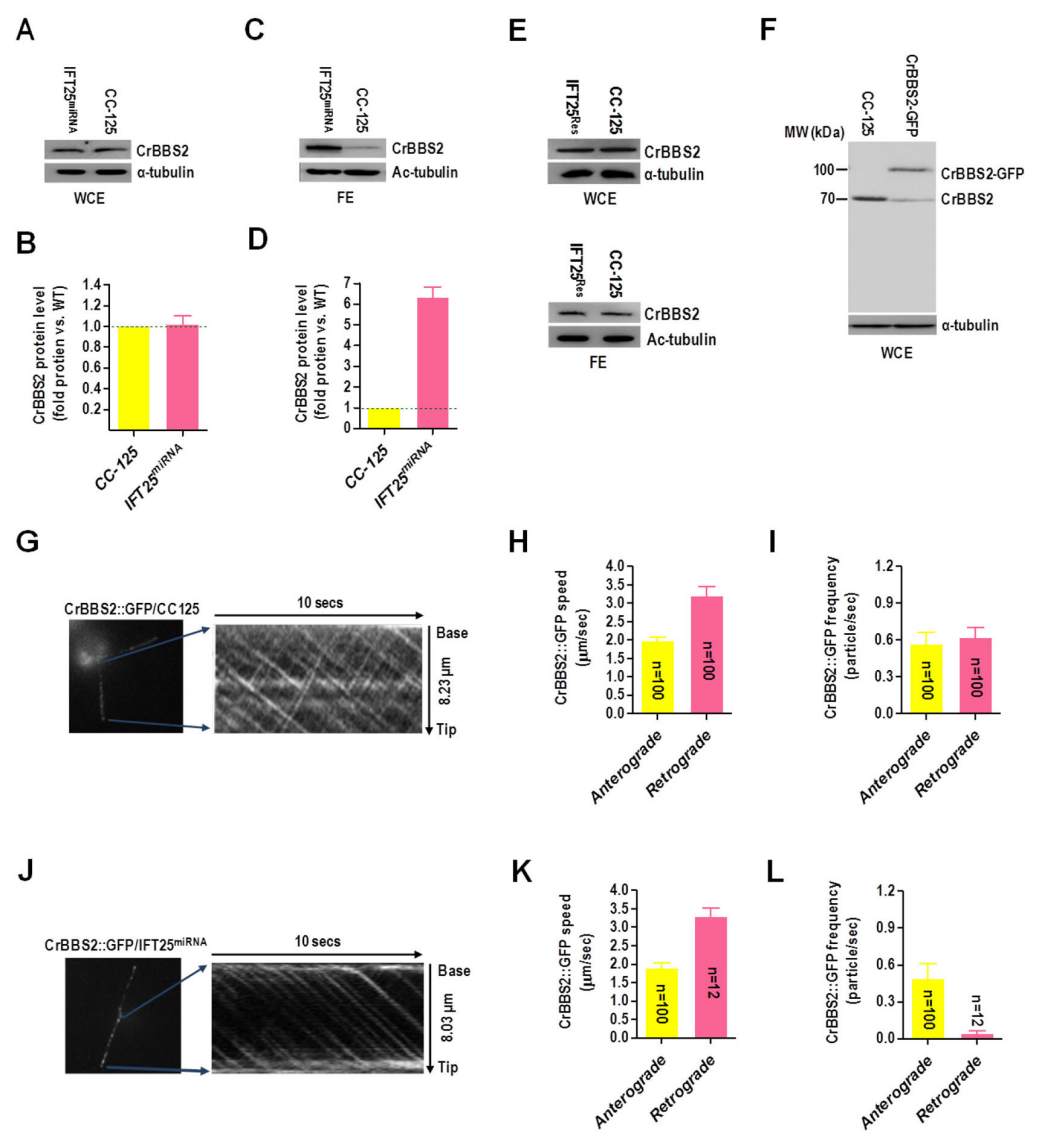
**IFT25 is required for the export of the BBSome from flagella.** (A) Western blots of wild-type CC-125 and IFT25^miRNA^ whole cell extract with α-CrBBS2. Alpha-tubulin was used as a western blot loading control. WCE, whole cell extract. (B) CrBBS2 protein levels were normalized to the α-tubulin housekeeping protein and presented as percentage-change relative to wild-type CC-125 CrBBS2. The data shown was calculated from three independent assays (*n*=3 repeats). Error bars indicate s.d. (C) Western blots of wild-type CC-125 and IFT25^miRNA^ flagella extracts with α-CrBBS2. Acetylated α-tubulin (Ac-tubulin) was used as a western blot loading control. FE, flagella extract. (D) CrBBS2 protein levels were normalized to the Ac-tubulin housekeeping protein and presented as percentage change relative to wild-type CC-125 CrBBS2. The data shown was calculated from three independent assays (*n*=3 repeats). Error bars indicate s.d. (E) Western blots of wild-type CC-125 and IFT25^Res^ whole cell and flagellar extracts with α-CrBBS2. Alpha-tubulin was used as a western blot loading control. WCE, whole cell extract; FE, flagellar extract. (F) Western blots of wild-type CC-125 cells and a CrBBS2-GFP-expressing CC-125 transgenic strain with antibodies to CrBBS2 and α-tubulin. (G) Single frame and kymograph from TIRF microscopy video imaging of CrBBS2::GFP transgenic strain (Movie 3, ∼10 fps). (H) The speeds of anterograde and retrograde CrBBS2::GFP in the CrBBS2::GFP transgenic strain. Anterograde CrBBS2::GFP speed is 1.98±0.10 μm/s (*n*=100, *P*=0.062) and retrograde CrBBS2::GFP speed is 3.21±0.23 μm/s (*n*=100, *P*=0.078). (I) The frequencies of anterograde and retrograde CrBBS2::GFP in the CrBBS2::GFP transgenic strain. The anterograde frequency of CrBBS2::GFP is 0.57±0.09 particle/sec (*n*=100, *P*=0.091) and its retrograde frequency is and 0.62±0.08 particle/sec (*n*=100, *P*=0.054). (J) Single frame and kymograph from TIRF microscopy video imaging of CrBBS2::GFP transgenic strain on an IFT25^miRNA^ cell background (Movie 4, ∼10 fps). (K) The mean speeds of anterograde and retrograde CrBBS2::GFP in the CrBBS2::GFP transgenic strain on an IFT25^miRNA^ cell background. Anterograde CrBBS2::GFP speed is 1.91±0.12 μm/s (*n*=100, *P*=0.013) and retrograde CrBBS2::GFP speed is retrograde CrBBS2::GFP speed is 3.30±0.23 μm/s (*n*=12, *P*=0.096) on an IFT25^miRNA^ cell background. (L) Mean frequencies of anterograde and retrograde CrBBS2::GFP in the IFT25^miRNA^ transgenic strain. The anterograde frequency of CrBBS2::GFP is 0.49±0.12 particle/sec (*n*=100, *P*=0.027) and its retrograde frequency is only 0.04±0.02 particle/sec (*n*=12, *P*=0.087). For panels H, I, K and L, speeds and frequencies are mean values±95% confidence interval and *P*-values were calculated from a student's *t*-test.

By generating a polyclonal antibody against the BBSome subunit, CrBBS2 (Fig. S12A,B), we identified that IFT25^miRNA^ cells contained CrBBS2 at a similar level as wild-type cells (Fig. 6A,B), indicating that IFT25 had no effect on the BBSome stability. However, the amount of CrBBS2 in the flagellum of IFT25^miRNA^ cells was dramatically increased as compared to that in wild-type cells (Fig. 6C,D). Given that the IFT25-expression recused IFT25^Res^ cells contained CrBBS2 both in whole cell and flagellar extracts at a similar level as wild-type cells (Fig. 6E), these results all together confirmed that the BBSome was accumulated in the flagellum due to IFT25 depletion. To measure its movement in flagella, a transgenic strain expressing CrBBS2::GFP was created (Fig. 6F). TIRFM analysis showed that CrBBS2::GFP fusion protein underwent typical bidirectional IFT (Fig. 6G; Movie 3). The anterograde and retrograde velocities of CrBBS2-GFP were determined to be 1.98±0.10 μm/s (*n*=100) and 3.21±0.23 μm/s (*n*=100), respectively, which all seem similar to that of other IFT proteins (Fig. 6H). This observation was in agreement with previous studies that the trafficking of the BBSome in flagella is an IFT-dependent process (Eguether et al., 2014; Lechtreck et al., 2009a; Liew et al., 2014; Ou et al., 2005; Williams et al., 2014). Frequencies of the anterograde and retrograde CrBBS2::GFP were measured to be 0.57±0.09 particle/sec (*n*=100) and 0.62±0.08 particle/sec (*n*=100), respectively (Fig. 6I). This was also in agreement with the previous observation showing that not all IFT particles were loaded with the BBSome complex in the flagellum of *C. reinhardtii* (Lechtreck et al., 2009a). To clarify if depletion of IFT25 can cause uncoupling of the BBSome from the IFT machinery a transgenic strain expressing CrBBS2::GFP fusion protein was created on an IFT25^miRNA^ cell background and the movement of the CrBBS2::GFP in the flagellum was measured by TIRFM. The kymograph showed that CrBBS2::GFP fusion protein underwent typical bidirectional IFT (Fig. 6J; Movie 4). Interestingly, although CrBBS2::GFP moved in an anterograde direction at a velocity of 1.91±0.12 μm/s (*n*=100), only a few retrograde-trafficking CrBBS2-GFP with normal speed (3.30±0.23 μm/s, *n*=12) were detected (Fig. 6K), showing that the retrograde movement of the BBSome was obviously impaired without IFT25. In addition, anterograde frequency of CrBBS2::GFP is 0.49±0.12 particle/sec (*n*=100), however its retrograde frequency is only 0.04±0.02 particle/sec (*n*=12), indicating that the retrograde CrBBS2-GFP was nearly undetectable (Fig. 6L). Taken together, these observations clearly showed that depletion of *C. reinhardtii* IFT25 leads to the hyper-accumulation of the BBSome in flagella by affecting its export from flagella.

## DISCUSSION

IFT particles in *C. reinhardtii* consist of two complexes, IFT-A (at least 6 subunits) and -B (at least 16 subunits) that separate in sucrose gradients (Cole et al., 1998; Fan et al., 2010; Follit et al., 2009; Ishikawa et al., 2014; Lechtreck et al., 2009b; Qin et al., 2007; Wang et al., 2009). Most recently, biochemical and recombinant reconstruction studies further divide IFT-B into two biochemically stable sub-complexes, the core IFT-B1 (IFT88/81/74/70/56/52/46/27/25/22) and the peripheral IFT-B2 (IFT172/80/57/54/38/20) (Katoh et al., 2016; Lucker et al., 2005; Taschner et al., 2016). Both sub-complexes act as potential IFT cargoes to carry tubulin/MT blocks and flagella signaling molecules necessary for the buildup of functional flagella (Taschner et al., 2016). Functional studies performed with a combination of several model organisms have shown that depletion of even a single core IFT-B1 subunit (Brazelton et al., 2001; Brown et al., 2015; Deane et al., 2001; Fan et al., 2010; Hou et al., 2007; Kobayashi et al., 2007; Pazour et al., 2000) can cause the complete disassembly of IFT-B, eventually leading to the occurrence of so-called ‘bald’ mutants. However, this is not always true because the core IFT-B1 subunit IFT56, once depleted, does not cause the instability of IFT-B and severe defects in flagella assembly (only slightly shorter flagella observed for IFT56 mutant) but instead leads to motility-associated defects of flagella in *C. reinhardtii* (Ishikawa et al., 2014) and hedgehog signaling defects in mouse (Swiderski et al., 2014). The core IFT-B1 subunits IFT25 and IFT27 are also not involved in maintaining the stability of IFT-B and flagellar assembly but required for the exchange of hedgehog signaling molecules and the BBSome between the cell body and flagella as tested in mouse model (Eguether et al., 2014; Keady et al., 2012). Taken together, the data in the literature showed that, even for some core IFT-B1 subunits, they are not structurally required for the maintenance of IFT-B stability and flagellar assembly but rather, more likely, act as IFT cargo proteins to carry flagella signaling molecules.

The IFT-B subunits IFT25 and IFT27 are unique among the IFT-B subunits since two proteins are not conserved in all ciliated organisms, suggesting that they are probably not required for flagellar assembly. Our previous *Chlamydomonas* study noticed that two proteins exist both in IFT-B complex and outside of it, demonstrating that IFT25 and IFT27 behavior is biochemically different from other IFT-B subunits (Wang et al., 2009). Two subsequent mouse studies showed that depletion of rodent IFT25 or IFT27 alone does not cause disruption of IFT particles and flagellar assembly but results in defects in signal-dependent movement of hedgehog components (Eguether et al., 2014; Keady et al., 2012). In this study, we identified that *Chlamydomonas* IFT25 and IFT27 form a heterodimeric complex *in vivo*, which physically exist both in IFT-B complex and outside of it. Both this study and other (Bhogaraju et al., 2011) *in vitro* studies confirmed that *Chlamydomonas* IFT25 is an obligate binding partner of IFT27 and its main function appears to protect IFT27 from aggregation. This notion was strengthened when knockdown of *Chlamydomonas* IFT25 was observed to eventually cause dramatic depletion of IFT27 *in vivo*. Different from the conventional IFT-B subunits, once depleted, typically causing the occurrence of ‘bald’ mutant, IFT25 depletion does not cause apparent disruption of IFT particles and the disassembly of flagella, indicating that IFT25 is independently structured within IFT-B. IFT25 was then uncoupled from other conventional IFT-B subunits in their function in flagella assembly. Interestingly, partial depletion of *Chlamydomonas* IFT27 had been reported to cause the instability of both IFT-A and -B and eventually lead to a complete absence of flagella and even defects in cytokinesis, suggesting that IFT27 is a critical factor required for not only IFT and flagella assembly but also for cell cycle control in *C. reinhardtii* (Qin et al., 2007). This could be true as IFT27 was probably not deprived to a level low enough to cause the reported phenotype in this study. However, *ift27* knockout in mouse and cultured cells also did not find its role in controlling IFT, flagella assembly and cell cycle (Eguether et al., 2014; Liew et al., 2014). Other than this, the specificity of the IFT27 knockdown phenotype has not been proven in *C. reinhardtii* in the previous study as a strict functional rescue assay was not performed and an off-target effect thus cannot be excluded (Qin et al., 2007).

*Chlamydomonas* and mouse BBSome is not required for IFT and flagellar assembly and its protein subunits are of relatively low abundance in flagella (Eguether et al., 2014; Keady et al., 2012; Lechtreck et al., 2009a). Disruption of the BBSome proteins causes dramatic accumulation of many signaling membrane proteins, indicating its role in coupling specific cargoes to the IFT machinery (Eguether et al., 2014; Keady et al., 2012; Lechtreck et al., 2009a). Mouse model has shown that depletion of IFT25/27 causes accumulation of the BBSome and signaling membrane proteins in the cilium, demonstrating that it is the IFT25/27 that acts as an adaptor between the BBSome and IFT machinery (Eguether et al., 2014; Keady et al., 2012; Liew et al., 2014). In this study, knockdown of *Chlamydomonas* IFT25 led to depletion of IFT27 and abnormal accumulation of the BBSome proteins in flagella. The BBSome accumulation in flagella is caused by disrupting the movement of the BBSome out of the flagella but not by interrupting the entry of the BBSome into the flagella, suggesting that IFT25/27 is an evolutionally conserved adaptor between the BBSome and IFT machinery across species. Together with the data shown in this study and the data in the literature (Aldahmesh et al., 2014; Eguether et al., 2014; Keady et al., 2012; Lechtreck et al., 2009a; Liew et al., 2014; Wang et al., 2009), we conclude that IFT25 behaves unlike a conventional IFT-B subunit and is dispensable for flagella assembly but required to export the BBSome from flagella in *C. reinhardtii*. Next we will further investigate if direct depletion of IFT27 can cause phenotype(s) the same as depletion of IFT25 in *C. reinhardtii*.

## MATERIALS AND METHODS

### Antibodies

Rabbit-raised polyclonal antibodies are shown in the supplementary Figs S1-7,9,10 and 12. Antibodies against HA (rat 3F10, Roche), GFP (mAbs 7.1 and 13.1, Roche), α-tubulin (mAb B512, Sigma-Aldrich) and Ac-α-tubulin (mAb 6-11B-1, Sigma-Aldrich) were commercially available. Rabbit-raised polyclonal antibody against *Chlamydomonas* IFT43 was reported previously (Zhu et al., 2017). For immunoblotting analysis, a dilution was used for IFT25 (1:500), IFT27 (1:200), IFT43 (1:1000), IFT46 (1:1000), IFT57 (1:1000), IFT72 (1:1000), IFT122 (1:1000), IFT139 (1:500), FLA10 (1:1000), D1BLIC (1:1000) and CrBBS2 (1:2000).

### Strains and culture conditions

*C. reinhardtii* wild-type strain CC-125 was used throughout this study and was obtained from the *Chlamydomonas* Genetic Center at the University of Minnesota, Twin Cities, MN, USA (www.chlamy.org). The IFT25 miRNA strain was generated as described below. For experiments, the strains were cultured in liquid or solid TAP medium at 23°C under continuous white light (∼80 μE/m^2^/s) with constant aeration when desired.

### Plasmids

Plasmids for the miRNA experiment were designed following a previously described method (Hu et al., 2014). Briefly, the miRNA targeting the *C. reinhardtii* IFT25 gene was designed using WMD3 software (http://wmd3.weigelworld.org). The output oligonucleotide was combined with the miRNA cre-MIR1157 (accession number MI0006219), resulting in a 167 bp IFT25 miRNA precursor sequence (atcaggaaaccaaggcgcgctagcttcctgggcgcagtgttccagctgcagtacGGGGGTAATGCCTAAGGATTActcgctgatcggcaccatgggggtggtggtgatcagcgctaTAATCCTTAGGCATTACCCCCtactgcagccggaacactgccaggagaatt). The sequences were synthesized by Genewiz (China), and ligated to the pHK263 plasmid (Hu et al., 2014). The resulting vectors were then named as pMi-IFT25. Expression vectors were created on a pBluescript KS(+) backbone and contained either HA::6×His or HA::GFP coding sequences followed immediately downstream by a sequence encoding the Rubisco 3′-UTR and the *aph*VIII cassette (paromomycin resistant gene). To express IFT25::HA::6×His and IFT25::HA::GFP, a 1.9 kb genomic DNA fragment composed of an 870 bp promoter sequence and the coding region of *ift25* was amplified from genomic DNA by using primer pair (5′-GGTGGATCCGGAAGATATGGCAAGCGTGC-3′ and 5′-GCTGAATTCGGCGTAGTCGGGCACGTCGTAGGGGTAGAACTCGTCCTCGAAGCCG-3′) and inserted into the corresponding expression vector. To generate IFT27::HA::6×His vector, a 2 kb IFT27 fragment consisting of the 1.2 kb promoter sequence and its coding region was amplified from genomic DNA by using primer pair 5′-GTACTAGTATGTACGACACTGCGTTAC-3′ and 5′-AAGAATTCGTAGTTGCGGCAAGCGACC-3′ and inserted into the corresponding expression vector. The rescue construct pBKS-gIFT25::HA::GFP-Ble was a derivative of pBKS-gIFT25::HA::GFP-Paro, created by replacing the *aphVIII* gene with a zeocin resistant gene (*ble*) amplified from pSP124S by suing primer pair 5′-AAGGGCCCGCCAGAAGGAGCGCAGCC-3′ and 5′-TTGGTACCGCTTCAAATACGCCCAGC-3′ (Stevens et al., 1996). To express CrBBS2::GFP, CrBBS2 cDNA were synthesized by Genewiz (China). The HSP70A-RBCS2 promoter was amplified from pBKS-gIFT25::HA::GFP-Paro by using primer pair 5′-GCTCTAGAGCCAGAAGGAGCGCAGCCAA-3′ and 5′-GCCATATGTTTAAGATGTTGAGTGACTTCT-3′. Then, the pBKS-HSP70A-RBCS2-CrBBS2::GFP-Paro and the pBKS-HSP70A-RBCS2-CrBBS2::GFP-Ble were constructed by replacing the gIFT25::HA::GFP DNA fragment in pBKS-gIFT25::HA::GFP-Paro and pBKS-gIFT25::HA::GFP-Ble with the CrBBS2::GFP fragment by a three-way ligation. The new constructs were verified by direct nucleotide sequencing. Details about constructs are available upon request.

### *Chlamydomonas* transformation

Transformation of the *C. reinhardtii* strain was performed using the electroporation method as described previously (Shimogawara et al., 1998). An exponential electric pulse of 2000 V/cm was applied to the cells using a Gene Pulser Xcell electroporation apparatus (Bio Rad). The capacitance was set at 50 μF and no shunt resistor was used. The transformants were selected on TAP plates with 20 µg/ml paromomycin (Sigma-Aldrich), 15 µg/ml bleomycin (Invitrogen), or both antibiotics. The plot showing the transformants and transformation efficiency was created with Prism 5 (GraphPad Software).

### SDS-PAGE and western blotting

Preparation of whole cell and flagellar extracts was performed as detailed previously (Wang et al., 2009), except that sonication method was instead used to open up the cell body and flagella. SDS-PAGE electrophoresis and western blotting was performed as previously described (Fan et al., 2010). If not otherwise specified, 10 µg of total protein from each sample was loaded for SDS-PAGE electrophoresis. To quantify the expression levels of proteins, the western blots were quantified with the histogram function in Mini Chemiluminescent Imaging apparatus software (Sage Creation) and defined as (MI_band_-MI_background_)*Pixels_band_. MI represents the mean intensity. MI_background_ is the mean intensity of the area around a target band. The intensity of the bands was normalized to the intensity of a loading control protein.

### DNA and RNA analysis

Genomic DNA was prepared using a Genomic DNA Prep kit following the kit's protocol (Solarbio, Beijing, China). Total cellular RNA was extracted from 1×10^8^ *C. reinhardtii* cells in the logarithmic phase of growth with Trizol reagent (Invitrogen) according to manufacturer's protocol. 5 µg of RNA from each sample was reverse transcribed at 42°C for 4 h followed by 95°C for 10 min using SuperScript^®^ III Reverse Transcriptase (Invitrogen) and oligo(dT)20 primers. Relative transcript amounts of specific IFT proteins were measured by SYBR-green quantitative PCR using primer pairs that span either introns or exons of the target genes with an A&B 7500 Fast Real-Time PCR System (Applied Biosystems). The PCR reactions were performed at 94°C for 2 min followed by 40 cycles of 94°C for 10 s, 60°C for 30 s, and 68°C for 2 min. Samples were normalized using guanine nucleotide-binding protein subunit beta-like protein (GBLP) as a housekeeping gene internal control. For each sample, three sets of mRNA were independently isolated and quantified three times each. Data was analyzed with GraphPad Prism 5 (GraphPad Software) and presented as mean±s.d. Primer pairs used were: 5′-AGTCTTGCATTCGGGTGTCT-3′ and 5′-CGAGCTCGACTTCAAAGACC-3′ for *ift25*; 5′-TCTCGGTGGAGCTCTTTCTG-3′ and 5′-GCTCACATCGAACACGAGAA-3′ for *ift27*; and 5′-GTCATCCACTGCCTGTGCTTCT-3′ and 5'-GGCCTTCTTGCTGGTGATGTT-3′ for GBLP.

### *In vivo* pull-down and gel filtration chromatography

*In vivo* pull-down of IFT25- and IFT27-associated protein complexes was performed using transgenic strains expressing C-terminal HA and 6×His double-tagged IFT25 and IFT27. The cells were lysed by sonication and the protein complex was purified by batch method using the Ni-NTA purification system (GE Healthcare) according to the company's protocol. 5 µg of each protein sample was analyzed by SDS-PAGE electrophoresis and western blotting as described above. To isolate IFT25/27, the eluate containing the Ni-NTA resin captured proteins was fractionated on a Superdex 200 10/300 GL column (GE Healthcare). Fractions (0.5 ml) were collected from the top of the gradient and subjected to SDS-PAGE electrophoresis and western blotting as described above. The molecular weight of the purified protein complexes were estimated by comparing to standard protein makers.

### Immunofluorescence microscopy

Immunofluorescence staining assay was performed as described in our precious study (Wang et al., 2009). Healthy sample cells grown under continuous illumination were seeded to 0.1% polyethyleneimine-coated coverslips for 8 min under strong light. The cells were then permeablized and fixed with ice-cold methanol twice each for 10 min. Thereafter, cells were rehydrated with phosphate buffered saline (PBS) and incubated overnight in blocking buffer (5% BSA, 1% cold fish gelatin, and 10% goat serum in PBS) at 4°C. The next day, primary antibodies were diluted in the blocking buffer and incubated with the coverslips for 4 h. After washing the cells ten times with PBS, Alexa-Fluor 488 conjugated goat anti-mouse IgG secondary antibody and Alexa-Fluor 542 conjugated goat anti-rabbit IgG secondary antibody (Molecular Probes, Invitrogen, USA) was diluted in the blocking buffer at a dilution of 1:200 and incubated with the coverslips for 2 h. After washing the cells an additional ten times with PBS, they were viewed on an Olympus BX53F fluorescent microscope at 400**×** amplification. Images were captured with an Olympus DP72 CCD camera (Olympus) with an exposure time between 0.002 and 2 s. The images were processed with ImageJ (version 1.42 g; NIH). The primary antibodies against IFT25 (1:5 dilution), IFT43 (1:50 dilution), IFT46 (1:50 dilution), GFP (1:5 dilution) have been described in the antibodies section above.

### Screening of the IFT25^miRNA^ cells and flagellar length analysis of the transgenic cells

The screening of IFT25^miRNA^ cells was initiated by checking the cellular level of the target protein through western blots of whole cell extracts with IFT25 antibody. IFT25^miRNA^ strains showing a reduced level of the target protein were selected for further phenotypic analysis. Measurement of flagellar length was performed as previously described (Fan et al., 2010). The data was processed with GraphPad Prism 5 (GraphPad Software). For each strain, over 50 cells were measured and flagellar lengths were presented as mean value (µm).

### Purification of bacterial-expressed proteins

A dual expression vector was created on a pGEX2T backbone (GE Healthcare) and three plasmids were generated for expression of IFT25 or IFT27 alone and for co-expression of IFT25 and IFT27. IFT25 and IFT27 were expressed with N-terminal GST (GST::IFT25) or 6×His (6×His::IFT27) tags. Bacterial expression of the recombinant proteins has been described previously (Fan et al., 2010). Clarified cell extract was applied to 0.4 ml glutathione-agarose beads (GST::IFT25), Ni-NTA resin (6×His::IFT27) or both for tandem purification of GST::IFT25 and 6×His::IFT27. Aliquots (8 μl) from each step of the purification were resolved on 10% SDS-PAGE gels and visualized with Coomassie Blue staining.

### Prokaryotic expression and purification of recombinant IFT protein antigens

Full-length *C. reinhardtii* IFT25, IFT46, IFT72 cDNAs and partial CrBBS2 (1-299 bp), IFT139 (1-459 bp) and IFT57 (955-1200 bp) cDNA fragments were cloned into pET-28a, pGEX-2T and pMAL-C2X vectors and resulted expression vectors were then expressed in *Escherichia* *coli* BL21(DE3) strain. Bacteria transformed with the desired plasmids were grown at 37°C in LB medium to an OD_600_ of 0.6, added 0.2 mM IPTG and reduced the growth temperature to 22°C to induce the protein expression for 6 h. For MBP tag purification: cells were collected, washed twice with PBS buffer (50 mM Tris-HCl, 500 mM NaCl, pH 7.4) and resuspended in 20 ml lysis buffer (20 mM Tris-HCl, 300 mM NaCl, 1 mM EDTA, 10 mM β-mercaptoethanol, pH 7.4), after being completely sonicated, centrifuged at 12,000 rpm/min (19802 x***g***) for 10 min, and the insoluble materials removed. The soluble proteins were then applied to 1 ml of Dextrin Sepharose™ High Performance column equilibrated with lysis buffer for 2 h at 4°C overnight and subsequently washed extensively by lysis buffer. At last, the bounded proteins were eluted from the resin using elution buffer (20 mM Tris, 300 mM NaCl, 1 mM EDTA, 10 mM β-mercaptoethano, 10 mM Maltose, pH 7.4). Samples were quantified with Brandford method and then subjected to SDS-PAGE electrophoresis and Coomassie Blue-staining. For 6×His tag purification: cells were collected, washed twice with PBS buffer (50 mM Tris-HCl, 500 mM NaCl, pH 7.4) and resuspended in 20 ml lysis buffer (50 mM Tris-HCl, 500 mM NaCl, 5 mM Imidazole, pH 7.4), being completely sonicated, centrifuged at 12,000 rpm/min (19802 x***g***) for 10 min, and removed the soluble buffer. The pellets were washed with lysis buffer (50 mM Tris-HCl, 500 mM NaCl, 5 mM Imidazole, pH 7.4) containing 2 M urea three times, and centrifuged at 12,000 rpm/min (19802 x***g***) for 10 min, and the insoluble materials collected. Pellets were dissolved with lysis buffer (50 mM Tris-HCl, 500 mM NaCl, 5 mM Imidazole, pH 7.4) containing 8 M urea, and were then applied to 1 ml of Ni Sepharose High Performance column equilibrated with lysis buffer for 2 h at room temperature or 4°C overnight and subsequently washed extensively by lysis buffer (pH 7.4). At last, the bounded proteins were eluted from the resin using elution buffer (50 mM Tris-HCl, 500 mM NaCl, 500 mM Imidazole, pH 7.4) containing 8 M urea. Samples were quantified with Brandford method and then subjected to SDS-PAGE electrophoresis and Coomassie Blue staining. For GST tag purification: cells were collected, washed twice with PBS buffer (50 mM Tris-HCl, 500 mM NaCl, pH 7.4) and resuspended in 20 ml lysis buffer (50 mM Tris-HCl, 500 mM NaCl, pH 7.4), after being sonicated completely, centrifuged at 12,000 rpm/min (19802 x***g***) for 10 min, and the insoluble materials removed. The soluble proteins were then applied to 1 ml of Glutathione Sepharose™ 4B High Performance column equilibrated with lysis buffer for 2 h at 4°C overnight and subsequently washed extensively by lysis buffer. At last, the bounded proteins were eluted from the resin using elution buffer (50 mM Tris-HCl, 300 mM NaCl, 2 mM MgCl_2_, 10 mM Glutathione, pH 7.4). Samples were quantified with Brandford method and then subjected to SDS-PAGE electrophoresis and Coomassie Blue staining.

### Preparation of polyclonal antibody

Two micrograms of purified-protein antigen, the N-terminal 17 amino acids (MVKKEVKPIDITATLRC) of IFT27, N-terminal 19 amino acids (MPPAGGGSESVKVVVRCRP) of FLA10, N-terminal 17 amino acids (MAAPAMLPGQAVKTPGS) of D1BLIC and N-terminal 19 amino acids (MRTVVAWQETPPEKDGVRN) of IFT122 (synthesized by ChinaPeptides, Shanghai, China) were mixed with Freund's Adjuvant (complete) (Sigma F5881, USA) for the first injection, and the subsequent two injections used Freund's Adjuvant (incomplete) (Sigma F5506, USA). The rabbits were raised in the animal service center of Tianjin University of Science and Technology and injected every 10 days. After collecting the antiserum, affinity purified with Protein A Sepharose™ CL-4B to specific bound IgG as per the manufacturer's instructions. Briefly, 1 ml antiserum was mixed with 14 ml binding buffer (12 mM Na_2_HPO_4_, 8 mM NaH_2_PO_4_, pH 7.0), and binding with Protein A resin equilibrated with binding buffer for 2 h at room temperature or 4°C overnight, washed the resin with binding buffer 3 times and eluted with elution buffer (0.1 M Glycine, pH 2.7). Experiments with rabbits are approved by Tianjin University of Science and Technology and performed according to the relevant regulatory guide for laboratory animals.

### IFT video imaging and speed measurements

The flagellar motility of GFP-tagged proteins was imaged at ∼10 frames per second (fps) using total internal reflection fluorescence (TIRF) microscopy on an inverted microscope (Eclipse Ti, Nikon) equipped with a through-the-objective TIRF system, a 100×/1.49 NA TIRF oil immersion objective (Nikon), and a cooled EM charge coupled device (CCD) camera (QuantEM:512SC, Photometrics) as detailed previously (Engel et al., 2009a,b). To quantify IFT speeds, kymographs were generated with Metamorph (version 7.7, MDS Analytical Technologies) and measured with NIS-Elements AR (version 3.2, Nikon) and ImageJ (version 1.42 g) as previously described (Engel et al., 2009a,b).

### Statistical analysis

All the data were presented as mean±s.d. Statistical analysis performed with GraphPad Prism 5 (GraphPad Software). For comparisons on speeds and frequencies of the GFP-labeled IFT proteins, one-sample unpaired *t*-test was used.

## Supplementary Material

Supplementary information

## References

[BIO026278C1] Aldahmesh M. A., Li Y., Alhashem A., Anazi S., Alkuraya H., Hashem M., Awaji A. A., Sogaty S., Alkharashi A., Alzahrani S. et al. (2014). IFT27, encoding a small GTPase component of IFT particles, is mutated in a consanguineous family with Bardet-Biedl syndrome. *Hum. Mol. Genet.* 23, 3307-3315. 10.1093/hmg/ddu044 24488770 PMC4047285

[BIO026278C2] Berbari N. F., Lewis J. S., Bishop G. A., Askwith C. C. and Mykytyn K. (2008). Bardet-Biedl syndrome proteins are required for the localization of G protein-coupled receptors to primary cilia. *Proc. Natl. Acad. Sci. USA* 105, 4242-4246. 10.1073/pnas.0711027105 18334641 PMC2393805

[BIO026278C3] Bhogaraju S., Taschner M., Morawetz M., Basquin C. and Lorentzen E. (2011). Crystal structure of the intraflagellar transport complex 25/27. *EMBO J.* 30, 1907-1918. 10.1038/emboj.2011.110 21505417 PMC3098482

[BIO026278C4] Bizet A. A., Becker-Heck A., Ryan R., Weber K., Filhol E., Krug P., Halbritter J., Delous M., Lasbennes M.-C., Linghu B. et al. (2015). Mutations in TRAF3IP1/IFT54 reveal a new role for IFT proteins in microtubule stabilization. *Nat. Commun.* 6, 8666 10.1038/ncomms9666 26487268 PMC4617596

[BIO026278C5] Brazelton W. J., Amundsen C. D., Silflow C. D. and Lefebvre P. A. (2001). The *bld1* mutation identifies the *Chlamydomonas osm-6* homolog as a gene required for flagellar assembly. *Curr. Biol.* 11, 1591-1594. 10.1016/S0960-9822(01)00485-7 11676919

[BIO026278C6] Brown J. M., Cochran D. A., Craige B., Kubo T. and Witman G. B. (2015). Assembly of IFT Trains at the ciliary base depends on IFT74. *Curr. Biol.* 25, 1583-1593. 10.1016/j.cub.2015.04.060 26051893 PMC4482480

[BIO026278C7] Cole D. G., Diener D. R., Himelblau A. L., Beech P. L., Fuster J. C. and Rosenbaum J. L. (1998). *Chlamydomonas* kinesin-II-dependent intraflagellar transport (IFT): IFT particles contain proteins required for ciliary assembly in *Caenorhabditis elegans* sensory neurons. *J. Cell Biol.* 141, 993-1008. 10.1083/jcb.141.4.993 9585417 PMC2132775

[BIO026278C8] Deane J. A., Cole D. G., Seeley E. S., Diener D. R. and Rosenbaum J. L. (2001). Localization of intraflagellar transport protein IFT52 identifies basal body transitional fibers as the docking site for IFT particles. *Curr. Biol.* 11, 1586-1590. 10.1016/S0960-9822(01)00484-5 11676918

[BIO026278C9] Dutcher S. K. (2003). Elucidation of basal body and centriole functions in *Chlamydomonas reinhardtii*. *Traffic* 4, 443-451. 10.1034/j.1600-0854.2003.00104.x 12795689

[BIO026278C10] Eguether T., San Agustin J. T., Keady B. T., Jonassen J. A., Liang Y., Francis R., Tobita K., Johnson C. A., Abdelhamed Z. A., Lo C. W. et al. (2014). IFT27 links the BBSome to IFT for maintenance of the ciliary signaling compartment. *Dev. Cell* 31, 279-290. 10.1016/j.devcel.2014.09.011 25446516 PMC4254547

[BIO026278C11] Engel B. D., Ishikawa H., Wemmer K. A., Geimer S., Wakabayashi K., Hirono M., Craige B., Pazour G. J., Witman G. B., Kamiya R. et al. (2012). The role of retrograde intraflagellar transport in flagellar assembly, maintenance, and function. *J. Cell Biol.* 199, 151-167. 10.1083/jcb.201206068 23027906 PMC3461521

[BIO026278C12] Engel B. D., Lechtreck K.-F., Sakai T., Ikebe M., Witman G. B. and Marshall W. F. (2009a). Total internal reflection fluorescence (TIRF) microscopy of *Chlamydomonas* flagella. *Methods Cell Biol.* 93, 157-177. 10.1016/S0091-679X(08)93009-0 20409817 PMC3686088

[BIO026278C13] Engel B. D., Ludington W. B. and Marshall W. F. (2009b). Intraflagellar transport particle size scales inversely with flagellar length: revisiting the balance-point length control model. *J. Cell Biol.* 187, 81-89. 10.1083/jcb.200812084 19805630 PMC2762100

[BIO026278C14] Fan Z.-C., Behal R. H., Geimer S., Wang Z., Williamson S. M., Zhang H., Cole D. G. and Qin H. (2010). *Chlamydomonas* IFT70/CrDYF-1 is a core component of IFT particle complex B and is required for Flagellar assembly. *Mol. Biol. Cell* 21, 2696-2706. 10.1091/mbc.E10-03-0191 20534810 PMC2912355

[BIO026278C15] Follit J. A., Xu F., Keady B. T. and Pazour G. J. (2009). Characterization of mouse IFT complex B. *Cell Motil. Cytoskeleton* 66, 457-468. 10.1002/cm.20346 19253336 PMC2753169

[BIO026278C16] Hou Y., Pazour G. J. and Witman G. B. (2004). A dynein light intermediate chain, D1bLIC, is required for retrograde intraflagellar transport. *Mol. Biol. Cell* 15, 4382-4394. 10.1091/mbc.E04-05-0377 15269286 PMC519134

[BIO026278C17] Hou Y., Qin H., Follit J. A., Pazour G. J., Rosenbaum J. L. and Witman G. B. (2007). Functional analysis of an individual IFT protein: IFT46 is required for transport of outer dynein arms into flagella. *J. Cell Biol.* 176, 653-665. 10.1083/jcb.200608041 17312020 PMC2064023

[BIO026278C18] Hu J., Deng X., Shao N., Wang G. and Huang K. (2014). Rapid construction and screening of artificial microRNA systems in *Chlamydomonas reinhardtii*. *Plant J.* 79, 1052-1064. 10.1111/tpj.12606 24974733

[BIO026278C19] Huet D., Blisnick T., Perrot S. and Bastin P. (2014). The GTPase IFT27 is involved in both anterograde and retrograde intraflagellar transport. *eLife* 3, e02419 10.7554/eLife.02419 24843028 PMC4003483

[BIO026278C20] Iomini C., Babaev-Khaimov V., Sassaroli M. and Piperno G. (2001). Protein particles in *Chlamydomonas* flagella undergo a transport cycle consisting of four phases. *J. Cell Biol.* 153, 13-24. 10.1083/jcb.153.1.13 11285270 PMC2185522

[BIO026278C21] Iomini C., Li L., Esparza J. M. and Dutcher S. K. (2009). Retrograde intraflagellar transport mutants identify complex A proteins with multiple genetic interactions in *Chlamydomonas reinhardtii*. *Genetics* 183, 885-896. 10.1534/genetics.109.101915 19720863 PMC2778984

[BIO026278C22] Ishikawa H., Ide T., Yagi T., Jiang X., Hirono M., Sasaki H., Yanagisawa H., Wemmer K. A., Stainier D. Y., Qin H. et al. (2014). TTC26/DYF13 is an intraflagellar transport protein required for transport of motility-related proteins into flagella. *eLife* 3, e01566 10.7554/eLife.01566. 24596149 PMC3936282

[BIO026278C23] Jin H., White S. R., Shida T., Schulz S., Aguiar M., Gygi S. P., Bazan J. F. and Nachury M. V. (2010). The conserved Bardet-Biedl syndrome proteins assemble a coat that traffics membrane proteins to cilia. *Cell* 141, 1208-1219. 10.1016/j.cell.2010.05.015 20603001 PMC2898735

[BIO026278C24] Katoh Y., Terada M., Nishijima Y., Takei R., Nozaki S., Hamada H. and Nakayama K. (2016). Overall architecture of the intraflagellar transport (IFT)-B complex containing Cluap1/IFT38 as an essential component of the IFT-B peripheral subcomplex. *J. Biol. Chem.* 291, 10962-10975. 10.1074/jbc.M116.713883 26980730 PMC4900248

[BIO026278C25] Keady B. T., Samtani R., Tobita K., Tsuchya M., San Agustin J. T., Follit J. A., Jonassen J. A., Subramanian R., Lo C. W. and Pazour G. J. (2012). IFT25 Links the Signal-Dependent Movement of Hedgehog Components to Intraflagellar Transport. *Dev. Cell* 22, 940-951. 10.1016/j.devcel.2012.04.009 22595669 PMC3366633

[BIO026278C26] Kobayashi T., Gengyo-Ando K., Ishihara T., Katsura I. and Mitani S. (2007). IFT-81 and IFT-74 are required for intraflagellar transport in *C. elegans*. *Genes Cells* 12, 593-602. 10.1111/j.1365-2443.2007.01076.x 17535250

[BIO026278C27] Kozminski K. G., Johnson K. A., Forscher P. and Rosenbaum J. L. (1993). A motility in the eukaryotic flagellum unrelated to flagellar beating. *Proc. Natl. Acad. Sci. USA* 90, 5519-5523. 10.1073/pnas.90.12.5519 8516294 PMC46752

[BIO026278C28] Lechtreck K.-F., Johnson E. C., Sakai T., Cochran D., Ballif B. A., Rush J., Pazour G. J., Ikebe M. and Witman G. B. (2009a). The *Chlamydomonas reinhardtii* BBSome is an IFT cargo required for export of specific signaling proteins from flagella. *J. Cell Biol.* 187, 1117-1132. 10.1083/jcb.200909183 20038682 PMC2806276

[BIO026278C29] Lechtreck K.-F., Luro S., Awata J. and Witman G. B. (2009b). HA-tagging of putative flagellar proteins in *Chlamydomonas reinhardtii* identifies a novel protein of intraflagellar transport complex B. *Cell Motil. Cytoskeleton* 66, 469-482. 10.1002/cm.20369 19382199 PMC2922027

[BIO026278C30] Liew G. M., Ye F., Nager A. R., Murphy J. P., Lee J. S., Aguiar M., Breslow D. K., Gygi S. P. and Nachury M. V. (2014). The intraflagellar transport protein IFT27 promotes BBSome exit from cilia through the GTPase ARL6/BBS3. *Dev. Cell* 31, 265-278. 10.1016/j.devcel.2014.09.004 25443296 PMC4255629

[BIO026278C31] Lin H., Nauman N. P., Albee A. J., Hsu S. and Dutcher S. K. (2013). New mutations in flagellar motors identified by whole genome sequencing in *Chlamydomonas*. *Cilia* 2, 14 10.1186/2046-2530-2-14 24229452 PMC4132587

[BIO026278C32] Lucker B. F., Behal R. H., Qin H., Siron L. C., Taggart W. D., Rosenbaum J. L. and Cole D. G. (2005). Characterization of the intraflagellar transport complex B core: direct interaction of the IFT81 and IFT74/72 subunits. *J. Biol. Chem.* 280, 27688-27696. 10.1074/jbc.M505062200 15955805

[BIO026278C33] Miller M. S., Esparza J. M., Lippa A. M., Lux F. G. III, Cole D. G. and Dutcher S. K. (2005). Mutant kinesin-2 motor subunits increase chromosome loss. *Mol. Biol. Cell* 16, 3810-3820. 10.1091/mbc.E05-05-0404 15944218 PMC1182318

[BIO026278C34] Mueller J., Perrone C. A., Bower R., Cole D. G. and Porter M. E. (2005). The FLA3 KAP subunit is required for localization of kinesin-2 to the site of flagellar assembly and processive anterograde intraflagellar transport. *Mol. Biol. Cell* 16, 1341-1354. 10.1091/mbc.E04-10-0931 15616187 PMC551497

[BIO026278C35] Niwa S. (2016). The nephronophthisis-related gene ift-139 is required for ciliogenesis in *Caenorhabditis elegans*. *Sci. Rep.* 6, 31544 10.1038/srep31544 27515926 PMC4981862

[BIO026278C36] Ou G., Blacque O. E., Snow J. J., Leroux M. R. and Scholey J. M. (2005). Functional coordination of intraflagellar transport motors. *Nature* 436, 583-587. 10.1038/nature03818 16049494

[BIO026278C37] Ou G., Koga M., Blacque O. E., Murayama T., Ohshima Y., Schafer J. C., Li C., Yoder B. K., Leroux M. R. and Scholey J. M. (2007). Sensory ciliogenesis in *Caenorhabditis elegans*: assignment of IFT components into distinct modules based on transport and phenotypic profiles. *Mol. Biol. Cell* 18, 1554-1569. 10.1091/mbc.E06-09-0805 17314406 PMC1855012

[BIO026278C38] Pazour G. J., Wilkerson C. G. and Witman G. B. (1998). A dynein light chain is essential for the retrograde particle movement of intraflagellar transport (IFT). *J. Cell Biol.* 141, 979-992. 10.1083/jcb.141.4.979 9585416 PMC2132779

[BIO026278C39] Pazour G. J., Dickert B. L. and Witman G. B. (1999). The DHC1b (DHC2) isoform of cytoplasmic dynein is required for flagellar assembly. *J. Cell Biol.* 144, 473-481. 10.1083/jcb.144.3.473 9971742 PMC2132917

[BIO026278C40] Pazour G. J., Dickert B. L., Vucica Y., Seeley E. S., Rosenbaum J. L., Witman G. B. and Cole D. G. (2000). *Chlamydomonas* IFT88 and its mouse homologue, polycystic kidney disease gene *tg737*, are required for assembly of cilia and flagella. *J. Cell Biol.* 151, 709-718. 10.1083/jcb.151.3.709 11062270 PMC2185580

[BIO026278C41] Pigino G., Geimer S., Lanzavecchia S., Paccagnini E., Cantele F., Diener D. R., Rosenbaum J. L. and Lupetti P. (2009). Electron-tomographic analysis of intraflagellar transport particle trains in situ. *J. Cell Biol.* 187, 135-148. 10.1083/jcb.200905103 19805633 PMC2762096

[BIO026278C42] Piperno G., Siuda E., Henderson S., Segil M., Vaananen H. and Sassaroli M. (1998). Distinct mutants of retrograde intraflagellar transport (IFT) share similar morphological and molecular defects. *J. Cell Biol.* 143, 1591-1601. 10.1083/jcb.143.6.1591 9852153 PMC2132975

[BIO026278C43] Porter M. E., Bower R., Knott J. A., Byrd P. and Dentler W. (1999). Cytoplasmic dynein heavy chain 1b is required for flagellar assembly in *Chlamydomonas*. *Mol. Biol. Cell* 10, 693-712. 10.1091/mbc.10.3.693 10069812 PMC25196

[BIO026278C44] Qin H., Wang Z., Diener D. and Rosenbaum J. (2007). Intraflagellar transport protein 27 is a small G protein involved in cell-cycle control. *Curr. Biol.* 17, 193-202. 10.1016/j.cub.2006.12.040 17276912 PMC1905864

[BIO026278C45] Rosenbaum J. L. and Witman G. B. (2002). Intraflagellar transport. *Nat. Rev. Mol. Cell Biol.* 3, 813-825. 10.1038/nrm952 12415299

[BIO026278C46] Shimogawara K., Fujiwara S., Grossman A. and Usuda H. (1998). High-efficiency transformation of *Chlamydomonas reinhardtii* by electroporation. *Genetics* 148, 1821-1828. 9560396 10.1093/genetics/148.4.1821 PMC1460073

[BIO026278C47] Stevens D. R., Rochaix J. D. and Purton S. (1996). The bacterial phleomycin resistance gene *ble* as a dominant selectable marker in *Chlamydomonas*. *Mol. Gen. Genet.* 251, 23-30. 8628243 10.1007/BF02174340

[BIO026278C48] Swiderski R. E., Nakano Y., Mullins R. F., Seo S. and Bánfi B. (2014). A mutation in the mouse *ttc26* gene leads to impaired hedgehog signaling. *PLoS Genet.* 10, e1004689 10.1371/journal.pgen.1004689 25340710 PMC4207615

[BIO026278C49] Taschner M., Weber K., Mourão A., Vetter M., Awasthi M., Stiegler M., Bhogaraju S. and Lorentzen E. (2016). Intraflagellar transport proteins 172, 80, 57, 54, 38, and 20 form a stable tubulin-binding IFT-B2 complex. *EMBO J.* 35, 773-790. 10.15252/embj.201593164 26912722 PMC4818760

[BIO026278C50] Tsao C.-C. and Gorovsky M. A. (2008). Tetrahymena IFT122A is not essential for cilia assembly but plays a role in returning IFT proteins from the ciliary tip to the cell body. *J. Cell Sci.* 121, 428-436. 10.1242/jcs.015826 18211962

[BIO026278C51] Wang Z., Fan Z.-C., Williamson S. M. and Qin H. (2009). Intraflagellar transport (IFT) protein IFT25 is a phosphoprotein component of IFT complex B and physically interacts with IFT27 in Chlamydomonas. *PLoS ONE* 4, e5384 10.1371/journal.pone.0005384 19412537 PMC2671599

[BIO026278C52] Williams C. L., McIntyre J. C., Norris S. R., Jenkins P. M., Zhang L., Pei Q., Verhey K. and Martens J. R. (2014). Direct evidence for BBSome-associated intraflagellar transport reveals distinct properties of native mammalian cilia. *Nat. Commun.* 5, 5813 10.1038/ncomms6813 25504142 PMC4284812

[BIO026278C53] Zhu B., Zhu X., Wang L., Liang Y., Feng Q. and Pan J. (2017). Functional exploration of the IFT-A complex in intraflagellar transport and ciliogenesis. *PLoS Genet.* 13, e1006627 10.1371/journal.pgen.1006627 28207750 PMC5336300

