# Supplementary material for: Chlamydomonas IFT25 is dispensable for flagellar assembly but required to export the BBSome from flagella
Source: Biol Open. 2017 Nov 15;6(11):1680–91. doi: 10.1242/bio.026278 (PMC5703605; doi:10.1242/bio.026278)
Supplement: Supplementary information [file biolopen-6-026278-s1.pdf]

Supplementary Figures

Fig. S1

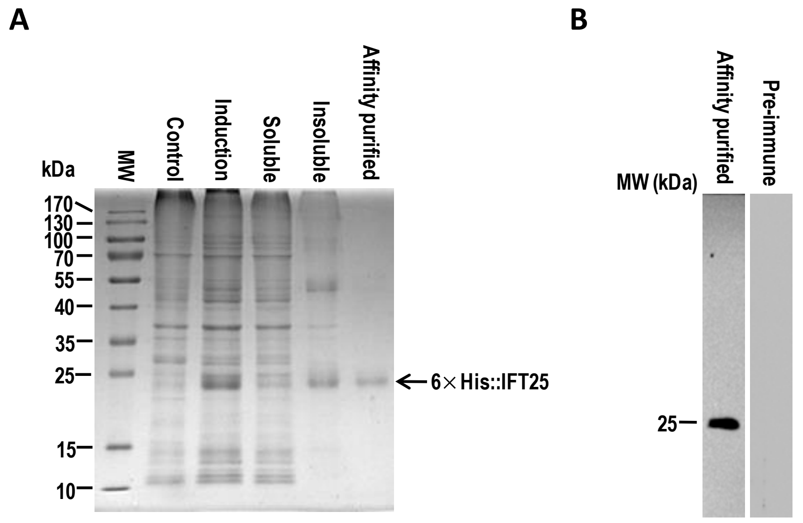

Fig. S1 6×His::IFT25 purification and western blots using the polyclonal anti-IFT25 antibody. (**A**) Purification of 6×His::IFT25 as an antigen for rabbit immunization. Coomassie Blue-stained gel shows that the bacterial-expressed recombinant 6×His::IFT25 is primarily present in the soluble fraction. The lanes indicated as "Control" and "Induction" contained the bacterial lysates before and after IPTG induction, respectively. The lanes labeled with "Insoluble" and "Soluble" are the insoluble and soluble fractions recovered after centrifugation of cell lysates. The lane labeled with "affinity purification" means the sample after the soluble fractions were further purified with Ni-NTA resin. (**B**) Whole cell extract of the wild-type CC-125 cells was probed with the affinity-purified IFT25 antiserum and a single band with a size of approximately 25 kDa was detected. In contrast, the preimmune serum does not recognize any specific band.

Biology Open • Supplementary information

Fig. S2

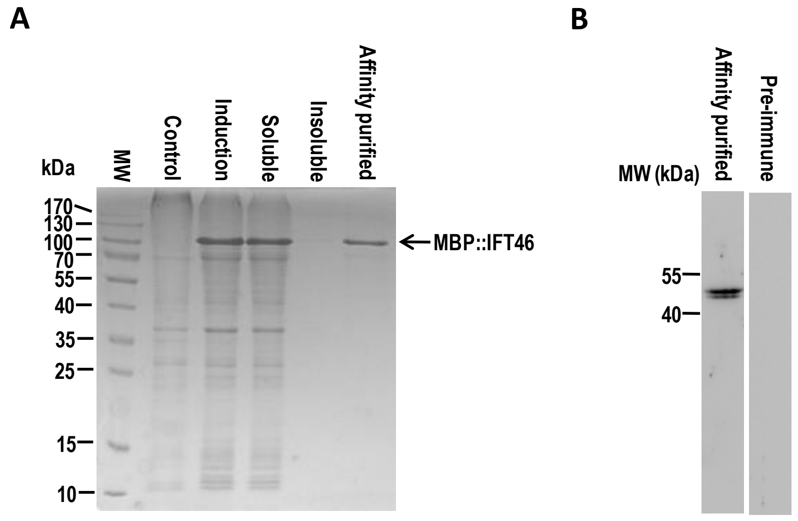

Fig. S2 MBP::IFT46 purification and characterization of the polyclonal anti-IFT46 antiserum in western blotting assay. (**A**) Purification of MBP::IFT46 as an antigen for rabbit immunization. Shown here is the Coomassie Blue-stained gel of samples from each step of the purification of the bacterial-expressed recombinant MBP::IFT46. The lanes indicated as "Control" and "Induction" contained the bacterial lysates before and after IPTG induction, respectively. The lanes labeled with "Insoluble" and "Soluble" are the insoluble and soluble fractions recovered after centrifugation of bacterial cell lysates. The lane labeled with "affinity purification" stands for the sample obtained after the soluble fractions of the bacterial cell lysates were further purified with amylase affinity resin. (**B**) Whole cell extract of the wild-type CC-125 cells was probed with the affinity-purified IFT46 antiserum and two bands which both have a size of approximately 46 kDa as expected were detected (14). In contrast, the pre-immune serum does not recognize any specific band.

Biology Open • Supplementary information

Fig. S3

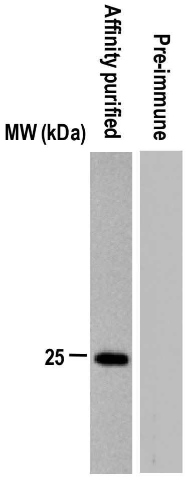

Fig. S3 Western blots with the polyclonal anti-IFT27 antiserum. Whole cell extract of the wild-type CC-125 cells was probed with the affinity-purified IFT27 antiserum and a single band with a size of approximately 20 kDa was detected. In contrast, the pre-immune serum does not recognize any specific band.

Fig. S4

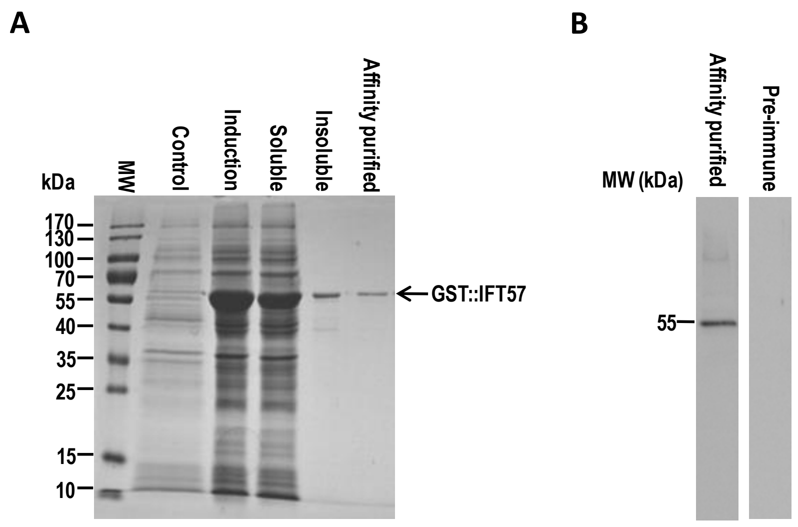

Fig. S4 GST::IFT57 purification and characterization of the polyclonal anti-IFT57 antiserum in western blotting assay. (**A**) Purification of GST::IFT57 (C-terminal 151 aa) as an antigen for rabbit immunization. Shown here is the Coomassie Blue-stained gel of samples from each step of the purification of the bacterial-expressed recombinant GST::IFT57. The lanes indicated as "Control" and "Induction" contained the bacterial lysates before and after IPTG induction, respectively. The lanes labeled with "Insoluble" and "Soluble" are the insoluble and soluble fractions recovered after centrifugation of bacterial cell lysates. The lane labeled with "affinity purification" stands for the sample obtained after the soluble fractions of the bacterial cell lysates were further purified with amylase affinity resin. (**B**) Whole cell extract of the wild-type CC-125 cells was probed with the affinity-purified IFT57 antiserum and one band which has a size of approximately 55 kDa as expected were detected. In contrast, the pre-immune serum does not recognize any specific band.

Biology Open • Supplementary information

Fig. S5

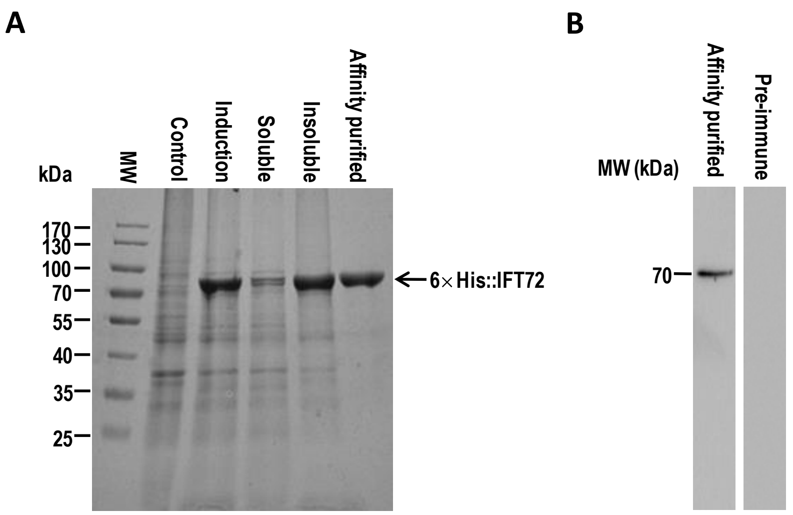

Fig. S5 6×His::IFT72 purification and characterization of the polyclonal anti-IFT72 antiserum in western blotting assay. (**A**) Purification of 6×His::IFT72 as an antigen for rabbit immunization. Shown here is the Coomassie Blue-stained gel of samples from each step of the purification of the bacterial-expressed recombinant 6×His::IFT72. The lanes indicated as "Control" and "Induction" contained the bacterial lysates before and after IPTG induction, respectively. The lanes labeled with "Insoluble" and "Soluble" are the insoluble and soluble fractions recovered after centrifugation of bacterial cell lysates. The lane labeled with "affinity purification" stands for the sample obtained after the soluble fractions of the bacterial cell lysates were further purified with amylase affinity resin. (**B**) Whole cell extract of the wild-type CC-125 cells was probed with the affinity-purified IFT72 antiserum and one band which has a size of approximately 70 kDa as expected were detected. In contrast, the pre-immune serum does not recognize any specific band.

Biology Open • Supplementary information

Fig. S6

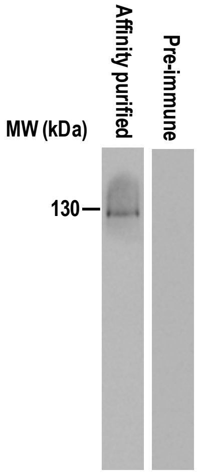

Fig. S6 Western blots with the polyclonal anti-IFT122 antiserum. Whole cell extract of the wild-type CC-125 cells was probed with the affinity-purified IFT27 antiserum and a single band with a size of approximately 120 kDa was detected. In contrast, the pre-immune serum does not recognize any specific band.

Biology Open • Supplementary information

Fig. S7

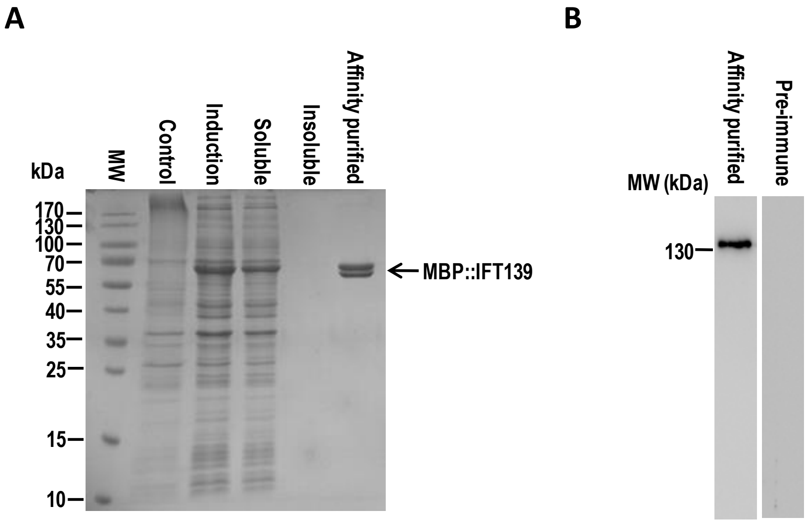

Fig. S7 MBP::IFT139 purification and Western blots with the polyclonal anti-IFT139 antiserum. (**A**) Bacterial-expressed MBP::IFT139 (N-terminal 153 aa) was affinity-purified as an antigen for rabbit immunization. Presented here is the Coomassie Blue-stained gel showing the samples from each step of the purification of the recombinant MBP::IFT139. The lanes indicated as “Control” and “Induction” contained the bacterial lysates before and after IPTG induction, respectively. The lanes labeled with “Insoluble” and “Soluble” are the insoluble and soluble fractions recovered after centrifugation of bacterial cell lysates. The lane labeled with “affinity purification” stands for the sample obtained after the soluble fractions of the bacterial cell lysates were further purified with amylase affinity resin. (**B**) Whole cell extract of the wild-type CC-125 cells was probed with the affinity-purified IFT139 antiserum and a single band with a size of approximately 139 kDa was detected. In contrast, the pre-immune serum does not recognize any specific band.

Biology Open • Supplementary information

Fig. S8

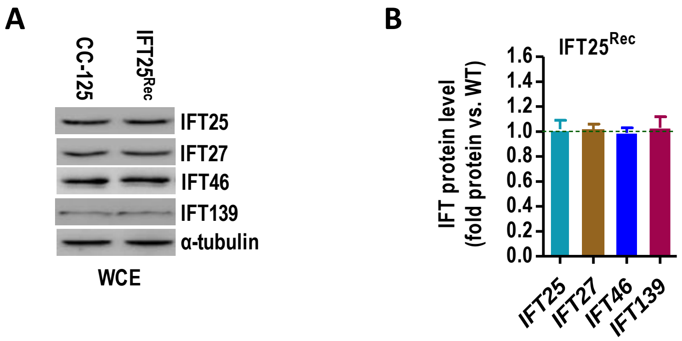

Fig. S8 IFT25 is essential to maintain IFT27 stability but not IFT-A and IFT-B *in vivo.* (**A**) Western blots comparing the cellular levels of IFT25, IFT27, IFT46 and IFT139 in wild-type CC-125 and the IFT25-expression recovered IFT25^{rec} cells. Alpha-tubulin was used as a Western blot loading control. (**B**) IFT protein levels were normalized to the α-tubulin housekeeping protein and presented as percentage-change relative to wild-type CC-125 IFT proteins. The data shown was calculated from three independent assays (n = 3 repeats). Error bars indicate SD. As calculated, IFT25^{rec} cells contained almost 100% as much IFT25, IFT27, IFT46 and IFT139 as CC-125 cells.

Biology Open • Supplementary information

Fig. S9

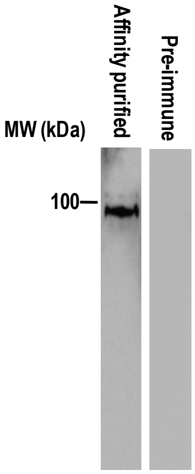

Fig. S9 Western blots with the polyclonal anti-FLA10 antiserum. Whole cell extract of the wild-type CC-125 cells was probed with the affinity-purified FLA10 antiserum and a single band with a size of approximately 90 kDa was detected. In contrast, the pre-immune serum does not recognize any specific band.

Biology Open • Supplementary information

Fig. S10

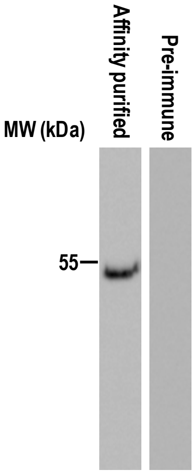

Fig. S10 Western blots with the polyclonal anti-D1BLIC antiserum. Whole cell extract of the wild-type CC-125 cells was probed with the affinity-purified D1BLIC antiserum and a single band with a size of approximately 50 kDa was detected. In contrast, the pre-immune serum does not recognize any specific band.

Biology Open • Supplementary information

Fig. S11

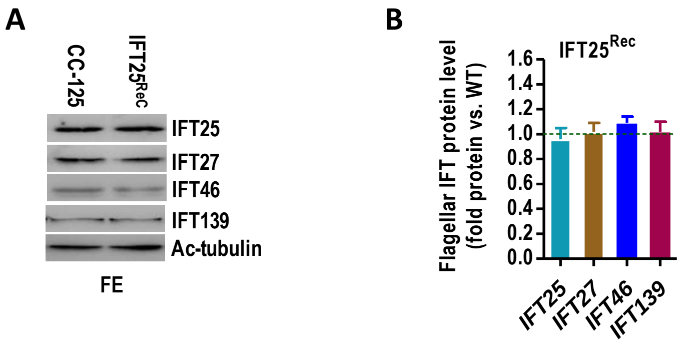

Fig. S11 IFT25 is essential to maintain the flagella level of IFT27 rather than IFT-A and IFT-B *in vivo*. (**A**) Western blots comparing the levels of IFT25, IFT27, IFT46 and IFT139 in flagella of wild-type CC-125 and the IFT25-expression recovered $IFT25^{rec}$ cells. Ac-tubulin was used as a Western blot loading control. (**B**) Flagella IFT protein levels were normalized to the Ac-tubulin housekeeping protein and presented as percentage-change relative to wild-type CC-125 IFT proteins. The data shown was calculated from three independent assays (n = 3 repeats). Error bars indicate SD. As calculated, $IFT25^{rec}$ cells contained IFT25, IFT27, IFT46 and IFT139 at a same level as CC-125 cells in flagella.

Biology Open • Supplementary information

Fig. S12

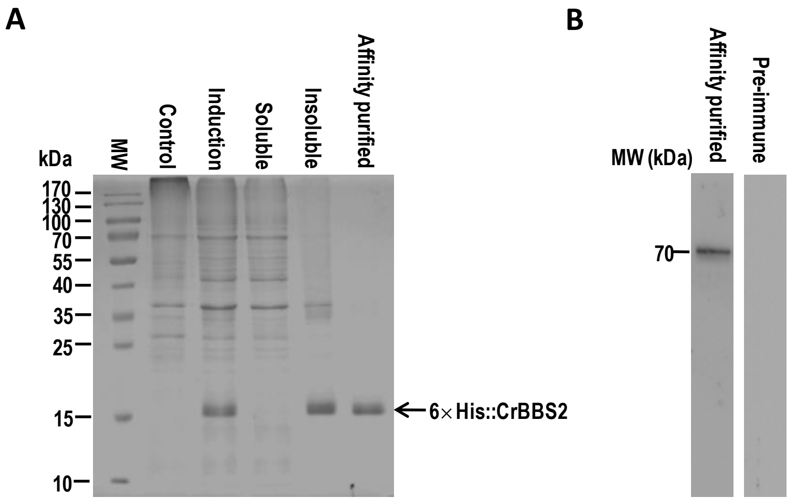

Fig. S12 6×His::CrBBS2 purification and Western blots with the polyclonal anti-CrBBS2 antiserum. (**A**) Bacterial-expressed 6×His::CrBBS2 (N-terminal 100 aa) was affinity-purified as an antigen for rabbit immunization. Shown here is the Coomassie Blue-stained gel of samples from each step of the purification of the recombinant 6×His::CrBBS2 protein. The lanes indicated as "Control" and "Induction" contained the bacterial lysates before and after IPTG induction, respectively. The lanes labeled with "Insoluble" and "Soluble" are the insoluble and soluble fractions recovered after centrifugation of bacterial cell lysates. The lane labeled with "affinity purification" stands for the sample obtained after the soluble fractions of the bacterial cell lysates were further purified with Ni-NTA resin. (**B**) The wild-type (wt) CC-125 whole cell extract was probed with the affinity-purified CrBBS2 antiserum and a single band with a size of approximately 70 kDa was detected. In contrast, pre-immune serum does not recognize any specific band

Biology Open • Supplementary information

Supplementary Movies

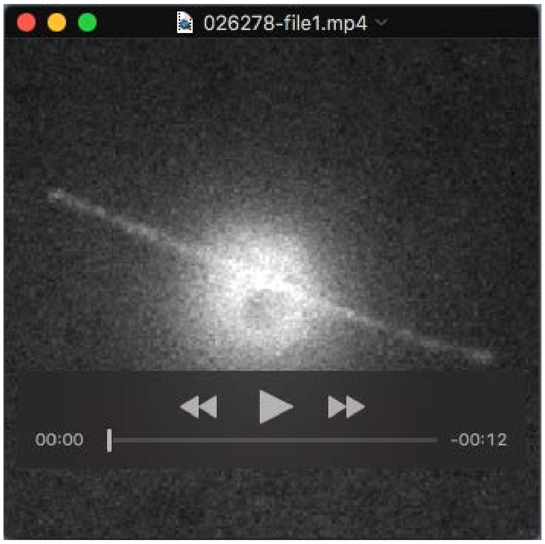

Movie S1. TIRF imaging of IFT25::HA::GFP movement in flagella of wild-type CC-125 strain. A frame from this movie and kymograph are shown in **Figure 1C**. Play speed is real-time (10 fps).

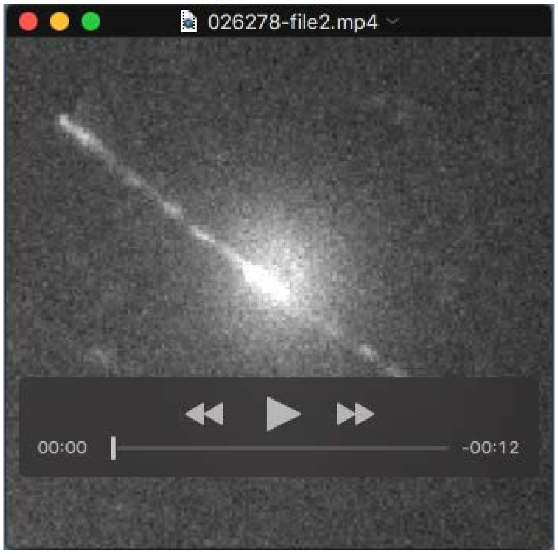

Movie S2. TIRF imaging of IFT25::HA::GFP movement in flagella of the IFT25^{miRNA} strain. A frame from this movie and kymograph are shown in **Figure 5H**. Play speed is real-time (10 fps).

Biology Open • Supplementary information

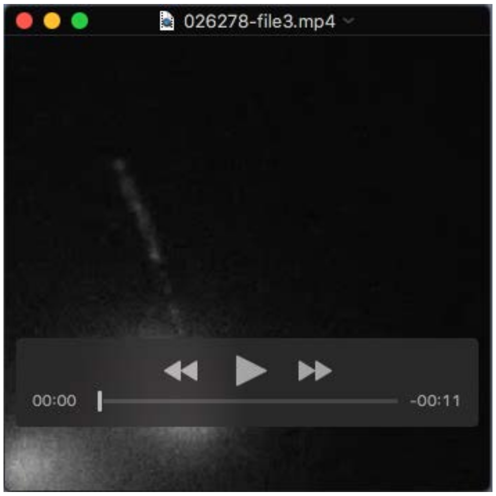

Movie S3. TIRF imaging of CrBBS2::HA::GFP movement in flagella of wild-type CC-125 strain. A frame from this movie and kymograph are shown in **Figure 6G**. Play speed is real-time (10 fps).

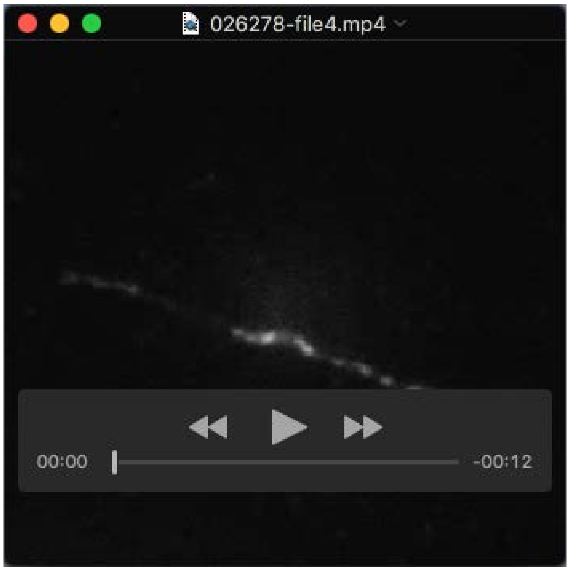

Movie S4. TIRF imaging of CrBBS2::HA::GFP movement in flagella of the $IFT25^{miRNA}$ strain. A frame from this movie and kymograph are shown in **Figure 6J**. Play speed is real-time (10 fps).

Biology Open • Supplementary information
